# Engineering of Bioresorbable Polymers for Tissue Engineering and Drug Delivery Applications

**DOI:** 10.1002/adhm.202401674

**Published:** 2024-09-04

**Authors:** Monika Dobrzyńska‐Mizera, Jagan Mohan Dodda, Xiaohua Liu, Monika Knitter, Reece N. Oosterbeek, Pablo Salinas, Eduardo Pozo, Ana Marina Ferreira, Emmanuel Rotimi Sadiku

**Affiliations:** ^1^ Institute of Materials Technology Polymer Division Poznan University of Technology Poznan Poland; ^2^ New Technologies – Research Centre (NTC) University of West Bohemia Univerzitní 8 Pilsen 30100 Czech Republic; ^3^ Chemical and Biomedical Engineering Department University of Missouri 1030 Hill Street Columbia Missouri 65211 USA; ^4^ Department of Engineering Science University of Oxford Parks Road Oxford OX1 3PJ UK; ^5^ Department of Cardiology Hospital Clínico San Carlos Madrid Spain; ^6^ Instituto de Investigación Sanitaria del Hospital Clínico San Carlos (IdISSC) Madrid Spain; ^7^ School of Engineering Newcastle University Newcastle upon Tyne Newcastle NE1 7RU UK; ^8^ Tshwane University of Technology Department of Chemical Metallurgical and Materials Engineering Polymer Division & Institute for Nano Engineering Research (INER) Pretoria West Campus Pretoria South Africa

**Keywords:** clinical challenges, drug delivery, implants, medical imaging, polymer matrices, resorbable biomaterials, tissue engineering

## Abstract

Herein, the recent advances in the development of resorbable polymeric‐based biomaterials, their geometrical forms, resorption mechanisms, and their capabilities in various biomedical applications are critically reviewed. A comprehensive discussion of the engineering approaches for the fabrication of polymeric resorbable scaffolds for tissue engineering, drug delivery, surgical, cardiological, aesthetical, dental and cardiovascular applications, are also explained. Furthermore, to understand the internal structures of resorbable scaffolds, representative studies of their evaluation by medical imaging techniques, e.g., cardiac computer tomography, are succinctly highlighted. This approach provides crucial clinical insights which help to improve the materials’ suitable and viable characteristics for them to meet the highly restrictive medical requirements. Finally, the aspects of the legal regulations and the associated challenges in translating research into desirable clinical and marketable materials of polymeric‐based formulations, are presented.

## Graphical abstract

This article provides a comprehensive overview of resorbable biomaterials, focusing on resorption mechanisms, fabrication methods, and their applications in medical devices. It explores innovations in dental tissue engineering, bone drug delivery systems, and regenerative surgery. Additionally, it discusses medical imaging techniques for bioresorbable devices, regulatory challenges, market potential, and future trends in the field.

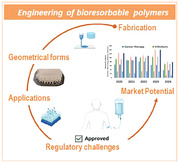

## 1 Introduction

Polymeric biomaterials, as defined by IUPAC, are “polymer‐based materials or polymer devices that are exploited in contact with living tissues, organisms, or microorganisms of therapeutic or biological interest”.^[^ ^1^ ^]^ Nowadays, they are gaining significant recognition in medical practice because of their useful utilization as appliances or their parts by physicians in various specializations. The developed polymeric medical grades with unique properties can be processed via the conventional plastic processing technologies and have been approved by the United States Food and Drug Administration (FDA).

Resorbable polymers are basically classified into two categories, natural and synthetic. The polymeric matrices of natural origin include polysaccharides (e.g., chitosan or alginate)^[^ ^2^, ^3^ ^]^ and proteins (e.g., collagen or keratin);^[^ ^4^, ^5^ ^]^ whereas, their synthetic counterparts (that have been in use for about half a century) include poly‐*ε*‐caprolactone (PCL), poly‐glycolic acid (PGA), poly(lactide) (PLA) or poly(d, l‐lactide) (PDLA) or their copolymers, e.g., poly(l‐lactide‐co‐d, l‐lactide) (PLDLLA), or poly(d, l‐lactide‐co‐glycolide) (PLGA).^[^ ^6^, ^7^, ^8^, ^9^, ^10^ ^]^ Of this list, there are representatives of biostable and bioresorbable polymers that find applications in tissue engineering with the aim to restore or replace sections or even the entire human tissues, such as: bones, cartilage, blood vessels, skin, etc.^[^ ^11^, ^12^ ^]^ The latter are particularly interesting since they serve as scaffolds to rebuild deficient tissue and are later eliminated from the body through natural pathways, i.e., kidneys via glomerular filtration or lungs after metabolization,^[^ ^1^ ^]^ thereby limiting the risk of short‐ and long‐term inflammatory responses. Polymeric material bio‐resorption, always exemplified by its biodegradation, must also be accompanied by other crucial features, such as biocompatibility and sufficient mechanical resilience.^[^ ^1^, ^7^ ^]^ The polymeric matrix can be tailored to a specific application by applying various types of modifications, including physical and chemical methods, e.g., the addition of osteoconductive ceramic fillers.^[^ ^13^ ^]^

Among various fillers, hydroxyapatite (HA, Ca_10_(PO_4_)_6_(OH)_2_) is a widely used bio‐ceramic filler for medical composites, since its chemical similarity to natural bone mineral (substituted with species including Na^+^, Mg^2+^, CO_3_^2−^, Cl^−^, ${\mathrm{HPO}}_{4}^{2_{-}}$) gives rise to good biocompatibility and bioactivity.^[^ ^14^ ^]^ Other crystalline calcium compounds, e.g., calcium carbonate and calcium orthophosphates have also found many uses in resorbable composites. The latter can be found in a wide range of crystalline and hydration states, including tetracalcium phosphate (TetCP), α‐tricalcium phosphate (α‐TCP), dicalcium phosphate dihydrate (DCPD), dicalcium phosphate anhydrous (DCP), octocalcium phosphate (OCP), and β‐tricalcium phosphate (β‐TCP).^[^ ^15^ ^]^ The non‐crystalline bio‐ceramics have become increasingly popular since the development of the first bioactive glass by Hench, a non‐crystalline silicate glass (45% SiO_2_, 24.5% Na_2_O, 24.5% calcium oxide (CaO), 6% phosphate (P_2_O_5_)) known as 45S5 or Bioglass.^[^ ^16^ ^]^ Other silicate‐based bioactive glass compositions^[^ ^17^, ^18^ ^]^ and P_2_O_5_‐based glasses have also become popular in recent years.^[^ ^19^, ^20^ ^]^ These often include CaO giving chemical similarities to HA and bone mineral, while modifications to the composition can give a wide variety of dissolution rates and biological responses.^[^ ^21^ ^]^

Tissue engineering is an interdisciplinary field that combines the principles learned from engineering and life sciences to develop biological substitutes that restore, maintain, or improve tissue function. This definition emphasizes the integration of various scientific disciplines to create functional replacements for damaged tissues and organs, such as skin, bones, cartilage, muscles (also cardiac), blood vessels, liver, as well as nervous, pancreatic, or corneal tissues, which is essential for the advancement of regenerative medicine.^[^ ^22^, ^23^, ^24^ ^]^ Bones, the most abundant hard tissues in the body, frequently suffer from diseases or fractures which require reconstruction; often ≈2M patients require bone crisis procedures, yearly.^[^ ^25^, ^26^ ^]^ Orthopedic surgeons typically use one of several different treatment methods, which can be categorized as: autografting (autologous bone), allografting and xenografting methods. Autografts, (cells and tissues grafted from one part to another of the same individual), are the most utilized since they possess osteogenic, osteoinductive, and osteoconductive features.^[^ ^27^, ^28^, ^29^ ^]^ Autografts may originate from different body regions; however, the most classically utilized is the iliac crest, since it contains a valuable source of progenitor cells and growth factors, its limits immune responses, it ensures ample bone amount and enables a comfortable harvesting procedure.^[^ ^30^ ^]^ Allografts, on the other hand, do not create a second surgical site since they are developed from processed bones, recovered from cadavers, or patients gaining hip joint prothesis, often in the form of cancellous bone.^[^ ^25^ ^]^ This solution provides an osteoconductive environment for vascularization and bone restoration; however, it may initiate immunological response or even convey contagious diseases.^[^ ^25^ ^]^ Xenografts involve the transmission of tissues from animals to humans, e.g., bovine bone that was previously subjected to deproteinization or sintering. The highest risk of transplantations between species, are associated with the recipient's immune rejection as well as possible xenogenic infections (xenozoonoses).^[^ ^30^ ^]^ The above‐mentioned imperfections may be overcome by the application of alloplasts, i.e., resorbable polymer‐based tissue substitutes; therefore, their future development as novel, and future‐oriented solutions is crucial.

Bioresorbable, alloplastic scaffolds serve as temporary devices that are expected to be overgrown with natural tissue, maintaining its shape and ensuring adequate mechanical support, and later on, removed systematically by the human body when no longer needed. First, a stable layer of autologous cells is formed, followed by the cell's proliferation, and differentiation.^[^ ^31^, ^32^ ^]^ Their greatest advantage in comparison to auto‐, allo‐, and xenografts, is their controlled resorption time. Three main factors that influence the resorption rate are: morphology, geometry, and the composition of the bioresorbable device.^[^ ^25^ ^]^ The morphology is expected to enhance the penetration of the growth factors inside the polymeric matrix in order to stimulate cell attachment, their migration and further development; therefore, porous materials (≤50 µm) or materials containing interspaces created as a result of the applied production process (e.g., Fused Deposition Modeling technology), are favored.^[^ ^25^, ^33^ ^]^ In addition, the resorption time may be controlled by various scaffold dimensions, i.e., thicker parts of the implant will generate longer resorption time. In the case of shape‐tailored scaffolds fitted to defective tissue, it is expected that the resorption time will vary as per the wall thickness; thereby, differentiating the period for the total implant disappearance. Other tactics involve the variation in the composition or structure of the material. The incorporation of sequences susceptible to degradation into polymeric backbone, greatly shortens the degradation time. Another effective route is to control the crystal structure of the polymer–amorphous structures are less resistant to resorption and known for limiting the risk of inflammation.^[^ ^34^, ^35^ ^]^ The addition of ceramic fillers in a composite greatly affects the ability of saturation of the composition with internal fluids; therefore, fastens the hydrolytic degradation of the material.^[^ ^34^ ^]^

In this review, an overview of the recent advances in the development of resorbable biomaterials, composites, their geometric forms, and capabilities in various biomedical applications, including tissue engineering, drug delivery, dental, and cardiovascular applications, is provided. A comprehensive discussion of the engineering approaches for the fabrication of resorbable composite scaffolds, bioresorbable stents, vascular grafts, and cardiac patches, which can offer clinical insights that can guide to improve the materials’ suitable characteristics has been carried out. Furthermore, in order to understand the internal structures of resorbable scaffolds, representative studies for the evaluation of bioresorbable scaffolds and stents by using cardiac computer tomography that can guide in the use of these scaffolds and the necessity to establish strategies for the design of multifunctional resorbable implants, for specific biomedical need, are succinctly highlighted.

## 2 Resorbable Biomaterials

Since the initial applications of bioresorbable polymers in areas such as sutures, a range of different synthetic polymer classes have been investigated.^[^ ^36^, ^37^, ^38^ ^]^ These are typically classified based on the type of bond present in the polymer backbone, which in turn has major effects on resorption mechanisms.^[^ ^39^ ^]^ Polyanhydrides have long been investigated, particularly for drug delivery applications, since their hydrophobicity and surface eroding resorption mechanism give rise to a near zero‐order drug release rate.^[^ ^40^ ^]^ Poly(ortho esters) tend to be even more hydrophobic, with a slower surface erosion rate, making them attractive for drug delivery, ophthalmic, and dental applications. Despite these advantages, wider investigation has been limited due the challenging requirements to synthesize and scale up these materials.^[^ ^41^, ^42^ ^]^ The most widely investigated class however, are poly(α‐hydroxy esters), with ester bonds in the polymer backbone that are hydrolyzed during resorption. Over the past decades, this class of bioresorbable polymers, which includes PLA, PGA, and PCL, has received extensive research interest.^[^ ^43^, ^44^ ^]^ The functionalization of these polymers is carried out by using end groups, such as OH^−^, Cl^−^, and NH_2_, or short polymer units of acrylate, fumarate, or ethylene glycol to tune biodegradation.^[^ ^45^, ^46^ ^]^ The use of other synthetic resorbable polymers, such as polypropylene carbonate (PPC) and polybutylene adipate terephthalate (PBAT), is not as widespread, but the utilization of such polymers, has been growing in recent years.^[^ ^47^, ^48^ ^]^ In addition, the polyhydroxylalkanoate (PHA) family, produced by bacterial fermentation, has received a considerable interest lately, e.g., polyhydroxybutyrate (PHB), polyhydroxyoctanoate (PHO), polyhydroxyvalerate (PHV).^[^ ^49^, ^50^, ^51^ ^]^ The copolymers of these mentioned polymers, e.g., poly(3‐hydroxybutyrate‐co‐3‐hydroxyvalerate) (PHBHV), are also attractive due to their non‐immunogenic, biocompatibility, and surface‐eroding degradation mechanism.^[^ ^52^ ^]^ Some other polymers, e.g., polybutylene succinate (PBS), can be produced via the natural (bacterial fermentation) and synthetic (petro‐based) methods.^[^ ^53^ ^]^ Several commercialized products from resorbable polymers are available in the market. For, e.g., surgical suture (Vicryl, 3‐0, Luxcryl PDO, Practimono PDO, Monoderm, Henry Schein, Auro thread, and Ethicon Stratafix), medical filament (Dioxactisse100, Glycolactisse85:15 and Caprolactisse100), oral and dental suture (Glyolon and Resorba), surgical meshes (Gliko and BioMesh), absorbable punctal plug for dry eye symptoms (Vera90, Oasis Soft Plug, ProLong, Extend, Comfortear Lacrisolve 180), and bioabsorbable extrusions (Absorv).

### 2.1 Resorption Mechanisms of Polymeric‐Based Implants

In the biomedical applications domain, the aqueous environment experienced by implant materials is the key driver of material degradation or resorption. Different constituent materials within resorbable composites degrade in various ways, however, the processes are not yet well understood. The following section outlines the current understanding of the degradation behavior and its effects on materials properties.

#### 2.1.1 Mechanisms of Resorption

As the most studied bioresorbable polymers, the degradation mechanisms of polyesters, e.g., PLA, are the most well understood. Initially water diffuses into the solid polymer according to the Fickian diffusion kinetics.^[^ ^54^ ^]^ Although this takes place from the outside, leading to the initial heterogeneous absorption; this is homogenized after a short time,^[^ ^55^ ^]^ when compared with the typical degradation times and thus for the PLA, this stage is usually ignored. The hydrolysis of the ester bonds occurs at both ends and by random chain scission,^[^ ^56^, ^57^ ^]^ leading to the production of oligomers with carboxylic end groups.^[^ ^58^ ^]^ The sample size is observed to influence the degradation mechanism, with large specimens limiting the mass transport of the reaction products out of the material, thereby causing a build‐up of acidic products and leading to autocatalysis of the hydrolysis reaction and hence, an accelerated degradation.^[^ ^55^, ^56^ ^]^ These autocatalytic effects are generally avoided in small devices which tend to be dominated by surface degradation.^[^ ^59^ ^]^ The reaction products are then consumed in the tricarboxylic acid cycle (Krebs cycle) to form CO_2_ and H_2_O, and then excreted.^[^ ^60^ ^]^ Some PGA degradation products can also be directly excreted by the kidneys, and enzymatic degradation is also known to play a role in the degradation of polyesters. The acidic degradation by‐products can cause the acidification of the local area around an implant, and in some cases, leading to inflammation. However, this has been reported to have a relatively low occurrence and typically, it only occurs in a poorly vascularized tissue, e.g., cartilage, where there is little fluid flow to dilute the degradation products.^[^ ^45^, ^61^, ^62^ ^]^ In addition to the polymers, viz: PLA, PGA, PCL, and their copolymers that have been the subject of research for some time, other resorbable polyesters, including PHAs, PPC, and PBS, are experiencing increasing and renewed interest. There has been limited systematic investigation into the mechanisms of their degradation, although they are known to differ significantly from the mechanisms of PLA and similar polymers. For instance, PHAs are hydrophilic and as a result, they degrade entirely by surface erosion rather than bulk degradation.^[^ ^52^ ^]^ An understanding of the degradation criterion of composites based on these polymers will therefore require detailed studies of the polymers’ degradation mechanisms.

In addition to the polymer phase, the material degradation of the inorganic filler phase (usually and specifically, dissolution) should be considered, and in this case the physical and chemical mechanisms are entirely different. The solubility of these materials depends on many physical and geometric factors, such as the particle size and density, but also their compositions and chemical structures. HA is relatively well characterized, demonstrating a very slow dissolution rate in vivo. Other crystalline forms of calcium phosphate^[^ ^15^ ^]^ increase the relative solubility in the order: TetCP ≈ *α*‐TCP > DCPD > DCP > OCP > *β*‐TCP > HA. By focusing on HA alone, there has been extensive work on the substituted HA with a range of cationic and anionic substitutions, both of which can alter the dissolution rate as well as the influence of the biological response to the material.^[^ ^14^ ^]^ Several dissolution models have been established to describe the dissolution of calcium phosphates,^[^ ^63^ ^]^ with homogeneous models that are suitable for simple HA, while the heterogeneous core‐shell models that incorporate a thin hydrated layer, are required for more complex systems, e.g., the substituted HA or nanocrystalline particles.^[^ ^64^ ^]^ Bioactive glasses, which have similar chemical components as bioceramics, e.g., HA, but are amorphous rather than crystalline, display widely variable dissolution rates, depending on their compositions. The original and most widely used bioglass, i.e., 45S5 (45% SiO_2_, 24.5% Na_2_O, 24.5% CaO, 6% P_2_O_5_), is known to form HA in vivo and a strong bond with bone—in this sense, it is resorbed into the body rather than dissolving.^[^ ^16^, ^65^ ^]^ Other glasses can display orders of magnitude with differences in their dissolution rates, in particular the phosphate glass system, which has attracted significant research interest for biomedical applications.^[^ ^21^, ^66^ ^]^ The dissolution of these glasses typically involves a stable surface layer formation, rich in glass network formers and depleted in the network modifying components.^[^ ^67^ ^]^ The formation of this surface layer depends on the glass composition and the surrounding fluid environment, and its stability has a profound impact on the dissolution rate of the glass. In recent years, multi‐stage models have been developed to describe the progression of glass dissolution that incorporates the formation and stabilization of this conversion layer.^[^ ^68^, ^69^, ^70^ ^]^ A key remaining challenge here, is the understanding of how the restricted environment within a polymer composite, affects the progression of the glass dissolution.

When bioresorbable polymers and inorganic fillers are combined to form composites, several additional factors that affect the material degradation are introduced. The size and morphology of the filler influences the extent of the wicking or interface effect, which is becoming increasingly recognized as a significant factor in composites degradation. Smaller particles with high surface area, increase the water absorption and therefore, degradation, as does with increase in the particles’ aspect ratios (i.e., short fibers), especially for continuous fiber reinforcement.^[^ ^71^, ^72^, ^73^ ^]^ The chemical composition of the filler also plays a key role. A buffering effect has been observed for certain ceramic fillers, where ion exchange occurs between protons in water and alkali ions in the glass/ceramic, resulting in a pH buffering effect at the glass surface and a reduced polymer degradation.^[^ ^74^, ^75^ ^]^ This effect can interact with the size effects, with smaller particles dissolving more quickly, and displaying a more effective buffering reaction.^[^ ^74^ ^]^ Kim et al., observed a reduction in the composite degradation rate in phosphate glass‐PCL composites with increasing CaO content in the glass. Increased CaO in phosphate glass is known to reduce the glass dissolution rate, however, this was also observed to have reduced the rate of polymer molecular weight reduction, which was attributed to the less acidic dissolution of high CaO‐containing glasses.^[^ ^76^ ^]^ Similar work by Mohammadi et al. on PCL composites with Si‐ or Fe‐substituted phosphate glasses did not observe this effect,^[^ ^77^ ^]^ suggesting that the polymer‐ceramic combination must be carefully selected in order to induce the acceleration of polymer degradation. The ability to control a composite degradation via glass composition is an attractive prospect that can decouple the degradation time from the mechanical properties, however, the full control of this event, is yet to be achieved.

#### 2.1.2 Influence of Resorption on Performance of Medical Devices

**Figure 1:**
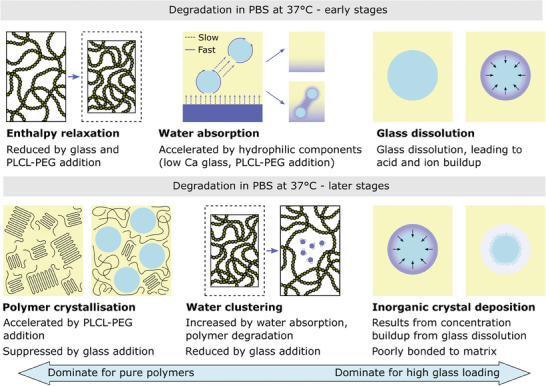
Schematic summary of the mechanisms of structural changes that occur in polymer‐glass composites during degradation, over different timescales, and for different filler loadings. Reproduced with permission.^[^ ^73^ ^]^

Historically, the changes in mechanical properties during material degradation have been relatively under‐investigated, despite the importance of the gradual transfer of load to the newly healed tissue. In recent years, this has been changing and more research is considering not just the initial mechanical properties and biodegradability, but also the evolution of the mechanical properties during this process.^[^ ^78^, ^79^ ^]^ In general, composites display superior mechanical properties before degradation when compared to the neat polymers, but these improved properties are often short‐lived and reduced rapidly, to the value below the neat polymer during degradation.^[^ ^80^, ^81^, ^82^ ^]^ This is usually a result of the water absorption scenario, occurring along the polymer‐filler interface which reduces mechanical integrity. The exact rate of this change in the material performance, is highly dependent on the components of the composite system; in particular, the polymer and filler surface chemistry (hydrophilicity), filler morphology and the amount of the filler inclusion. Other structural changes can occur during degradation, in particular in the polymer phase where mobile polymer chains can rearrange (by aging or crystallization), which in some cases can lead to the strengthening of the composite, in the early stages of degradation.^[^ ^83^ ^]^ Recent work^[^ ^73^ ^]^ has begun to provide a more comprehensive view of the mechanisms of structural changes experienced during composite degradation and its effect on material properties (**Figure** **1**), but further work is needed to expand this phenomenon in order to encompass the broad range of polymer and inorganic phase compositions that are commonly used.

Changes in the composite mechanical properties during degradation can also be understood from a fundamental perspective by computational modeling. The models are based on the foundational work of Pan et al. describing simultaneous molecular weight reduction and crystallization of bioresorbable polymers during degradation,^[^ ^84^ ^]^ and the linking of this to the mechanical properties by using a constitutive law.^[^ ^85^ ^]^ Such models are now used to describe the degradation of medical devices in vivo.^[^ ^86^ ^]^ Despite the advantages of being able to predict the evolution of the mechanical properties of a material during degradation, attempts to extend these models to bioresorbable composites, have been limited. Pan et al. extended their polymer model to include calcium phosphate dissolution and the resulting buffering effect,^[^ ^87^ ^]^ while Kobayashi and Yamaji focused on the role of ceramic particles in increasing the water content.^[^ ^88^ ^]^ Moreno‐Gomez^[^ ^89^ ^]^ developed a generalized modeling framework, based on the work of Pan et al. that could then be particularized to describe the degradation of composites containing TCP, HA, and calcium carbonate. Despite these recent advances, further work in this area is necessary to bridge the gap between experimental observations and phenomenological models to accurately capture the range of mechanisms experienced by the variety of different composites, including a wider range of polymers, and fillers. This should enable the prediction of not just the mass loss and molecular weight reduction in the polymer, but also the evolution of the mechanical properties during degradation.

### 2.2 Fabrication of Resorbable Medical Devices

The fabrication of resorbable composite materials based on the traditional composite processing techniques often requires various adaptations to protect the sensitive nature of degradable materials. Melt and solvent processing methods are widely used,^[^ ^90^, ^91^, ^92^ ^]^ with several recent implementations for resorbable implants. In recent years, additive manufacturing has also been of a particular focus, since it has been the subject of significant research efforts.^[^ ^93^, ^94^ ^]^ The ability to produce engineered porous architectures and the potential for the design of patient‐tailored implants, make additive manufacturing ideal for many biomedical applications.

#### 2.2.1 Thermal/Melt Processing

**Figure 2:**
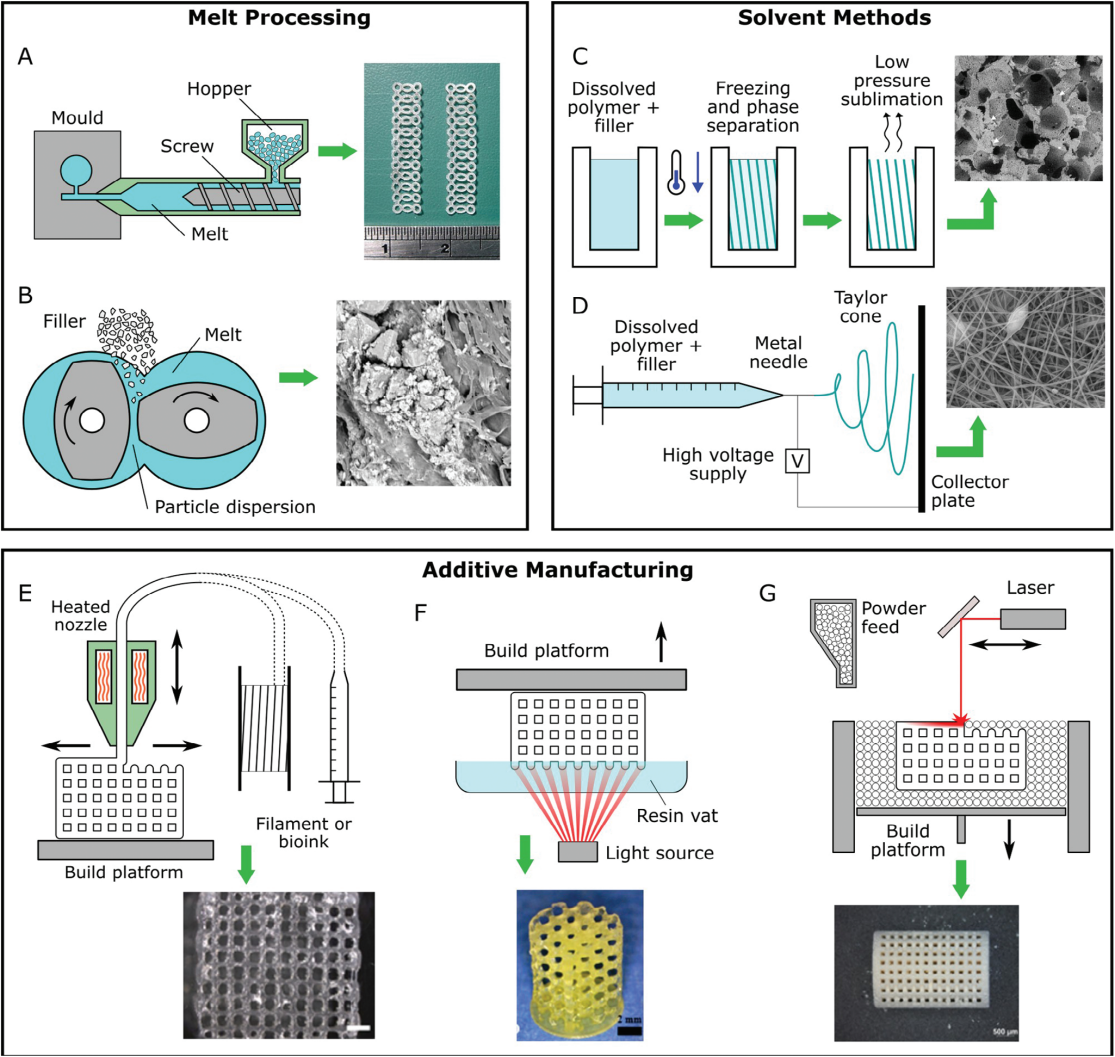
Illustrations of common fabrication techniques for bioresorbable polymers and composites. Melt processing: A) injection molding or micro‐injection molding, B) melt compounding (twin‐screw extrusion); Solvent processing: C) freeze drying, D) electrospinning; and Additive manufacturing: E) materials extrusion or direct ink writing, F) vat photopolymerization, and G) powder bed methods like powder bed fusion or binder jetting. Reproduced with permission^[^ ^95^, ^96^, ^97^, ^98^, ^99^, ^100^ ^]^ and under CC BY‐NC‐ND 4.0.^[^ ^101^ ^]^

The melt processing methods, such as injection molding and extrusion (**Figure** **2**A), are widespread across many industries, including the medical and biomedical domains. Their advanced stage of development makes the melt processing methods to be highly scalable from the pilot scale up to industrial production, where large volumes of parts can be made quickly and at a relatively low cost. Despite their popularity, they have several drawbacks, including being limited to relatively simple device geometries without intricate features. The large scale, equipment cost, and amount of material required, pose further obstacles to their use in research and development of bioresorbable composites. More recently, the microinjection molding has gained popularity, however, the high cost of such equipment is still a limitation, leading to the development of low‐cost pilot‐scale injection molding systems.^[^ ^102^ ^]^

The greatest challenge in the melt processing of polymeric composites is ensuring a good filler dispersion within the matrix. The traditional melt blending process, uses the screw rotation approach to mix the ingredients (Figure 2B). This exposes the polymer to high temperatures and shear forces, which may cause premature polymer degradation.^[^ ^103^, ^104^ ^]^ This is a particular problem for resorbable polymers, e.g., polyesters, which are vulnerable to hydrolysis, initiated by absorbed water.^[^ ^105^ ^]^ In addition, polyesters are sensitive to reaction with bioactive glass at high temperature, leading to a significant reduction in the molecular weight and mechanical properties.^[^ ^106^, ^107^ ^]^ This is believed to occur by thermal catalysis of the reaction between the SiO^−^ species in the glass, and the ester groups in the polymer, thereby forming carboxylate salts and oligomer fragments.^[^ ^105^, ^106^ ^]^ Sarasua et al. employed the plasma surface modification of the bioactive glass to hinder this reaction and prevent degradation during processing, however, this technique requires further optimization and additional processing steps.^[^ ^106^ ^]^

Another notable melt processing technique for resorbable composites is the fiber mat stacking, employed by Ahmed et al.^[^ ^71^, ^108^, ^109^, ^110^, ^111^ ^]^ This method is akin to the lay‐up of conventional fiberglass that is ubiquitous in construction materials, but instead uses PLA and melt‐spun phosphate glass fibers. The capability to selectively orient the glass fibers to provide reinforcement in certain directions is attractive, however, the technique has not been widely adopted for bioresorbable composites due to the extensive water wicking along the continuous fibers, which in turn leads to the deterioration of mechanical properties and accelerated degradation of the resulting composite.

#### 2.2.2 Solvent Processing

The solvent processing methods involve dissolving a polymer in a suitable organic solvent, mixing it with the filler, if needed, thereby, shaping the composite, and finally evaporating solvent (Figure 2C). This method can be employed to produce a composite feedstock for further processing by thermal methods, e.g., injection molding, hence, avoiding the excessive thermal‐ and shear‐induced degradation associated with an additional high‐temperature mixing step before molding. This method is popular due to its simplicity and lack of expensive equipment requirements. Such materials have been used as feedstocks for further thermal molding.^[^ ^112^, ^113^, ^114^ ^]^ The main disadvantage of solvent casting method, involves the filler sedimentation that occurs during drying leading to particles’ agglomeration and mechanical properties’ deterioration.^[^ ^115^ ^]^ Possible solutions include the alteration of the matrix viscosity^[^ ^116^, ^117^ ^]^ or the usage of a solvent‐solvent precipitation method^[^ ^115^ ^]^ to reduce the tendency for aggregation and the creation of large defects that could initiate early fracture.

Another drawback of the solvent‐based methods is the solvent itself. Some commonly used solvents, e.g., dichloromethane, chloroform, are toxic and potentially carcinogenic, leading to safety concerns over their usage in the production of biomedical implants. Although they are intended to be completely removed, residual solvent has been found to be retained within polymer films at levels of ≈10 wt.%.^[^ ^118^ ^]^ Being not only a toxicity concern, but it also affects the mechanical properties of the materials.^[^ ^118^, ^119^, ^120^ ^]^ Residual solvent may act as a plasticizer or lead to a reduction in the PLA tensile strength by >60%.^[^ ^118^ ^]^

Electrospinning is an established fabrication method for polymers, wherein ultrafine fibers can be spun from solutions by using a strong electric field that produces a randomly oriented or aligned mat^[^ ^61^, ^121^ ^]^ (Figure 2D). Inorganic particles can also be added to the polymer solution to enable a composite production, typically by using nanoparticles of bioactive glass, resulting in enhanced mechanical properties, improved biological performance, and in some cases, antibacterial properties.^[^ ^121^ ^]^ Ao et al. examined the electrospun composites of cellulose and HA nanoparticles. Electrospinning was able to fabricate fiber diameter distributions comparable to those of natural extracellular matrix (ECM) fibers, in the range of between 100 and 200 nm, with high strength and good biocompatability.^[^ ^122^ ^]^ The pore size distribution in electrospun fiber mats is another key parameter that governs their biocompatibility. Typical electrospun mats have pore sizes in the order of several microns, and up to tens of microns. For bone tissue engineering purposes, the optimal pore size is thought to be approximately 300 µm, which is significantly larger than that produced by electrospinning. However, the loose bonding of electrospun fiber mats is thought to allow cell migration by mechanical interaction with the material, expanding the size of the pore.^[^ ^123^ ^]^ Recent work by Liverani et al. successfully produced electrospun composites of PCL/chitosan/bioactive glass by using more benign, non‐toxic solvents (acetic acid, formic acid), showing a potential pathway to alleviating the negative effects of the solvent processing method, mentioned above.^[^ ^101^ ^]^

Thermally induced phase separation (TIPS), or freeze‐drying, is another technique that enables the production of porous composite materials, however when compared with the additive manufacturing technique (see below) the structure cannot be designed on a pore‐by‐pore basis. TIPS freezes the solvent/polymer/filler mixture, and then reduces the pressure to sublime the solvent and leaves a highly porous scaffold. In contrast to many other composite production methods, the incorporation of filler particles (up to 15 wt.%) into porous scaffolds, produced by TIPS, has minimal effect on the scaffold mechanical properties. It was observed to be more effective in imparting bioactivity than it was in controlling the mechanical or degradation behaviors.^[^ ^124^, ^125^ ^]^ A more recent study by Szustakiewicz et al.,^[^ ^126^ ^]^ investigated scaffolds with very high HA content, and began to observe a significant increase in the Young's modulus, once the HA content reached 75 wt.%. The key advantage of incorporating inorganic fillers into polymer scaffolds produced by TIPS is the biological effect, with the ion release from PCL/biomineral scaffolds, encouraging apatite nucleation for bone regeneration,^[^ ^127^ ^]^ and other formulations, based on PLLA/bioactive glass demonstrating the ability to maintain osteochondral cell phenotype for articular cartilage repair.^[^ ^128^ ^]^

#### 2.2.3 Additive Manufacturing

Developments in additive manufacturing (AM) technology have enormous potential for the fabrication of resorbable composites for biomedical applications. The accurate control over material macro‐ and micro‐structures, provided by AM, offers many possibilities for resorbable composite scaffolds in areas, such as bone or soft tissue scaffolds (Figure 2E–G). The main modes^[^ ^129^, ^130^ ^]^ of AM, include powder bed fusion (PBF), binder jetting (BJT), material extrusion (MEX), material jetting (MJT), and vat photopolymerization (VPP).

**Figure 3:**
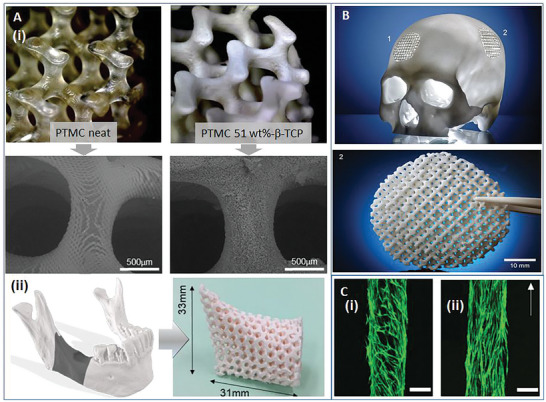
A) (i) SEM images of the scaffolds produced by using the VPP, showing the neat polymer (with smooth surface), and composites with *β*‐TCP (with microscale surface roughness). (ii) Example of the route to produce a porous and personalized composite implant for a large mandibular defect. Reproduced with permission.^[^ ^131^ ^]^ B) Example of a fully resorbable cranial implant manufactured by the PBF technique from a composite of PLA and calcium carbonate. Reproduced with permission^[^ ^132^ ^]^ under the CC‐BY 4.0 license. C) A magnetically assisted orientation of fibers in a composite produced by the DIW technique, showing cell orientations on the composites with: random (i) and aligned (ii) fibers. Scale bar = 250 µm. Reproduced with permission.^[133.]^

The PBF is mainly used for permanent medical implants, however, it has recently been extended to resorbable composite materials.^[^ ^134^ ^]^ It is well suited for composite fabrication due to the ease of tailoring the composition by the simple mixing of powders. Gatto et al. employed the PBF technique to produce fully resorbable porous composite scaffolds from PCL and HA, enabling the design and control of macro‐porosity into specific unit cell geometries, which determined the mechanical properties and influenced biological response.^[^ ^135^ ^]^ Several similar works^[^ ^132^, ^136^ ^]^ were also used to establish bioresorbable polymers and ceramics, such as PLA, HA, and calcium carbonate (**Figure** **3**), which are known for favorable biological responses for bone regeneration applications. Recent works are taking advantage of the versatility of material selection, with Wang et al. incorporating bioactive glass into poly‐ether‐ether‐ketone (PEEK) to improve the material stiffness and promote mineralization.^[^ ^137^ ^]^ PBF, taking advantage of the high resolution, was initially limited to engineering thermoplastics, e.g., nylon, however, these early examples have demonstrated a shift towards resorbable polymers and composites for medical implants.

VPP (also known as digital light processing, DLP) is emerging as a fabrication technique for resorbable composites due to its exceptional resolution that can be down to ≈10 µm, enabling the construction of highly complex scaffolds. With the photopolymerization method, a key technical challenge is the availability of polymer resins that are both photopolymerisable and resorbable. The polymers used in VPP include PCL,^[^ ^138^, ^139^ ^]^ PLA,^[^ ^140^ ^]^ poly(propylene fumarate),^[^ ^141^ ^]^ and  poly(l‐lactide‐co‐ε‐caprolactone).^[^ ^142^ ^]^ Saed et al. used functionalized PLA with biphasic calcium phosphate (BCP), by successfully incorporating ≈45 wt.% BCP in additively manufactured scaffolds, while resins with higher proportions of BCP were too viscous for successful printing.^[^ ^143^ ^]^ Dienel et al. used the VPP to fabricate composites by using poly(trimethylene carbonate) and *β*‐TCP.^[^ ^131^ ^]^ Up to 60 wt.% *β*‐TCP was successfully incorporated into printed scaffolds. This provided a significant mechanical reinforcement, and the bioactive agent (*β*‐TCP) was observed on the external surface, in contrast to the melt processing technique, where polymer forms a skin over the entire surface. This immediate availability of the bioactive agent is another advantage of the VPP. The achievement of an appropriate resin viscosity can be challenging when using the VPP, however composite paste has been used to successfully fabricate polyethylene glycol diacrylate (PEGDA) composites with ≈40 wt.% HA.^[^ ^144^ ^]^ To polymerize the resin, a photoinitiator must be used to catalyze the curing process; these are often industrial curing agents like TPO (diphenyl(2,4,6‐trimethylbenzoyl)phosphine oxide) or similar compounds, which can demonstrate cytotoxicity.^[^ ^145^ ^]^ There is thus a focus on developing new, more biocompatible photoinitiators with suitable photochemical properties, such as TPA‐DTP (2,6‐bis(triphenylamine)dithieno[3,2‐b:2′,3′‐d]phosphole oxide).^[^ ^146^ ^]^ Future works need to focus on the degradation of these new materials, photopolymerization side effects, and the effective parameters for the process. For instance, recent work of Herwig et al. using poly(orthoester‐thioether) (POETE), considered not only the photopolymerization and cytocompatibility, but also characterized the degradation rate and surface‐eroding degradation mechanism.^[^ ^147^ ^]^

Within the AM, the BJT technique is still an emerging technology, however some recent implementations have demonstrated its potential for resorbable composite materials. As a powder‐based process, it has traditionally been popular for ceramics. This includes Bioglass and *β*‐TCP as recently demonstrated by Bose et al.^[^ ^100^ ^]^ Such ceramic scaffolds may be later infiltrated with a polymeric matrix, as demonstrated by Ahn et al. by the melt infiltration of 40 vol.% PCL into a BCP scaffold.^[^ ^148^ ^]^ Inzana et al. demonstrated an alternative method that incorporated a resorbable polymer (collagen) into the binder solution during the BJT of calcium phosphate.^[^ ^149^ ^]^ This takes advantage of the low‐temperature nature of the BJT process to use a more thermally sensitive biopolymer. A different approach was employed by Dini et al. who used a mixture of HA and polymer powders (carboxymethyl chitosan, polyvinylpyrrolidone, and dextrin) to print composites with 60 wt.% HA.^[^ ^150^ ^]^ From these examples, it is obvious that the BJT method is a promising method for production of composites with higher ceramic content (≥60 wt.%), which are well suited to hard tissue applications in bone regeneration.

The MJT technique has an important role in the production of resorbable implant materials. This method is popular for bioprinting where live cells are included to create living bioactive materials due to low temperatures and liquid‐based processing. Despite the shear stresses applied, cell viability can often be maintained.^[^ ^151^ ^]^ However, the utilization of this method for resorbable composites is challenging since the incorporation of ceramic particles increases the viscosity, leading to nozzle clogging and difficulties in ejecting the droplets. As a result, to date, few works have been carried out in this area.^[^ ^152^ ^]^ By using inkjet printing Gao et al. were able to successfully print composites of polyethylene glycol dimethacrylate with HA, bioactive glass, and human mesenchymal stem cells.^[^ ^153^ ^]^ The inclusion of these inorganic phases increased the mechanical properties and improved osteogenic differentiation, applicable for both hard and soft tissues, however their low elastic modulus (typically ≤100 kPa) makes them unsuitable for load‐bearing applications.

There are two main sub‐types of MEX relevant for resorbable materials, fused deposition modeling (FDM) and direct ink writing (DIW). FDM is a ubiquitous method that applies melting and solidification of thermoplastic filament, while DIW typically uses viscoelastic inks (polymer liquids or gels) that flow easily through the nozzle under shear, forming rigid structures once the shear stress is removed. These inks can include living cells, but the viscosity requirements are less stringent, and hence composite production is more viable. These composite inks are intensively researched as detailed in the review by Heid and Boccaccini.^[^ ^152^ ^]^ Although these materials show great potential, further improvement in their mechanical properties and more in‐depth knowledge of filler‐matrix interactions are still required. Recently, works have begun by Heid et al. to control a composite degradation, demonstrating tuneable crosslinking of printed hydrogels by the addition of calcium silicate,^[^ ^154^ ^]^ and the work of Allen et al. showing that HA addition to hydrogels, decreased swelling and resisted enzymatic degradation.^[^ ^155^ ^]^ Novel materials, e.g., hydrogels containing nano‐attapulgite for improved mechanical properties, and greater osteogenic and angiogenic responses,^[^ ^156^ ^]^ or the composite hydrogels that contained fibers, functionalized with magnetic iron oxide nanoparticles, enabling fiber alignment to mimic tissue anisotropy,^[^ ^133^ ^]^ are still in their emerging stages. Resorbable composites produced by FDM have also been the subject of extensive research efforts.

In recent years, filament extrusion systems have become available at a small scale, enabling the formulation of novel composites for FDM containing a range of resorbable materials, such as PLA, PCL, HA, TCP, and bioactive glass.^[^ ^157^, ^158^, ^159^, ^160^ ^]^ Inorganic filler contents of ≈10 wt.% are common, resulting in an improved bioactivity. Improvements in mechanical properties are not always observed with the inclusion of inorganic filler particles, often leading to stress concentrations and reduced toughness when compared with pristine polymers. Although these composites often display superior mechanical properties, and when compared to the softer gel‐based composites, they are frequently insufficient for load‐bearing medical applications, therefore, addressing this concern remains valid. It is obvious that the modern manufacturing techniques, especially the AM methods, have given scientists and engineers the unparalleled ability to produce resorbable composites with highly controllable structures and compositions, at a range of length scales. A complete understanding of how the processes affect the degradation behavior, and the complex evolution of mechanical properties upon degradation is however, lagging.^[^ ^161^ ^]^ More importantly, it is desirably needed to translate advances in the fabrication capabilities into actual medical outcomes.

### 2.3 Geometrical Forms of Currently Utilized Implants

Amongst all the biomedical applications, bone substitutions are the most often applied; therefore, implants for their reconstruction display the widest variety. Before the process of tissue reconstruction may begin, the polymeric biomaterial must be appropriately shaped, as detailed above. This is to ensure a close matching between the implant and defective tissue.^[^ ^162^ ^]^ There are several approaches that are currently under consideration for tissue engineering, namely, the standardized products, devices with undefined geometrical structures, and the implants that are personalized in terms of their shapes and compositions. The latter approach is of the highest interest bearing future‐oriented solutions that are adjustable to each patient's needs. All the approaches are summarized herein.

#### 2.3.1 Standardized Implants

**Figure 4:**
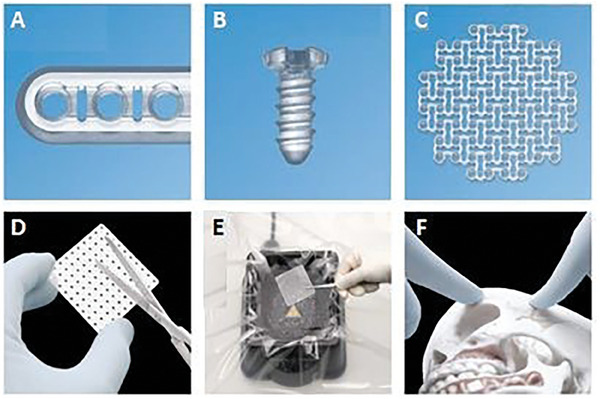
RapidSorb fixation system for maxillofacial procedures: A) plate, B) screw, C) mesh/foil, D) adjustment of implant size during operation, E) water bath, and F) adjustment of implant shape during operation. Reprinted from [178116.pdf (llnwd.net)].

The standardized medical devices are mainly used in oral, maxillofacial, and orthopedic surgeries, for the fixations of fractures (trauma) as well as securing autografts, osteotomies, orthognathic procedures, and many more.^[^ ^163^ ^]^ These three‐dimensional systems include a wide variety of screws, plates, meshes, and membranes (**Figure** **4**A–C) and they are frequently based on PLA, PGA, or their copolymers and doped with ceramic fillers, e.g., hydroxyapatite.^[^ ^163^, ^164^ ^]^ These types of implants are usually manufactured by using the standard polymer processing methods, e.g., injection molding. Because of the varieties in the geometries, sizes, and compositions of these implants, their biodegradation kinetics may be controlled and adjusted from weeks to years.^[^ ^164^ ^]^

These types of systems are quite universal since the manufacturers provide a large diversity of sizes, e.g., different screw diameters and lengths. However, these solutions have a form of flat surface and often, do not correspond to the curvature of damaged bone, especially in the case of maxillofacial surgeries. Therefore, the medical procedure must foresee additional preparation steps during the surgery. Firstly, the implant size must be adjusted according to the patient's needs (Figure 4D). Thereafter, the appropriate curvature needs to be obtained and in the case of naturally stiff PLA, it is not a straightforward procedure. It is, therefore, necessary to heat up a mesh or plate above its glass transition temperature^[^ ^165^ ^]^ (usually ≈60 °C); therefore, in the surgical site, water baths are used to increase the implant temperature that is later hand‐shaped (Figure 4E,F). This procedure extends the applicability of such systems, however, it is also time‐consuming, increases the risk of implant contamination, and depends on the medical staff's experience and skills.

#### 2.3.2 Products with Undefined Geometry

For medical cases where geometrically standardized products cannot be utilized, for instance, in the case of open, undefined, or relatively minute defects, solutions with undefined geometries are preferred. These solutions, include granules of different types, usually with a diameter of ≈5 mm, porous blocks, cement pastes, putties, and recently developed hydrogel‐based formulations. These may be categorized as materials in solid or liquid states.^[^ ^25^ ^]^

The first class includes granules and blocks that were successfully used for open defects with easy access. The granules may be composed of human or animal (usually bovine) deproteinized or sintered bone, which limits the biological risks that are related to organic contamination.^[^ ^25^, ^166^ ^]^ They induce bone formation throughout the entire defect; however, they exhibit negligible mechanical strength and often migrate even through the stitched wounds during convalescence. Porous blocks, made of allografts or polymeric materials, can alternatively be used for specified defect shapes, e.g., rectangular, or cylindrical, with an open access. An effective usage of such materials has been already studied in vivo with promising results.^[^ ^167^, ^168^, ^169^ ^]^ Like granules, sponges can form a mature bone throughout the entire damage with a negligible mechanical support for the defect. Because of the compact form, migration during and after handling is limited, however, some difficulties may arise to adjust the shape to the defective site.^[^ ^25^ ^]^

The second class of bone substitutes, includes materials that are injectable due to their liquid or paste form. Hydraulic cements usually contain osteoconductive calcium phosphate particles and they undergo hardening processes upon the addition of aqueous solutions,^[^ ^25^, ^170^ ^]^ leading to the physical entanglement of crystals and their mutual inter‐growth. Putties, on the other hand, do not yield curing processes since they consist of hard particles, often allograft or ceramic, dispersed within viscous binding matrix. A range of putties are now approved and available for clinical use. These often include bioactive glass as a filler, ensuring osteoconductive properties, and collagen or polyethylene glycol as the polymeric matrix. Commercially available products,^[^ ^20^ ^]^ include NovaBone, Medpor‐Plus, Bonalive, and OssiMend Bioactive. This injectable class creates a non‐invasive method to pack defects with limited access or even in closed spaces. However, this ability may be limited by the high viscosity of the material or the premature hardening process.^[^ ^25^ ^]^ Recently, a new class of injectable hydrogels for tissue regeneration was developed, often with chitosan, alginate, or hyaluronic acid‐based, which are able to undergo sol–gel reaction, induced by either the body temperature upon implementation (thermosensitive) or pH‐induced gelation (pH sensitive)^[^ ^171^, ^172^, ^173^, ^174^ ^]^

#### 2.3.3 Customized Products

**Figure 5:**
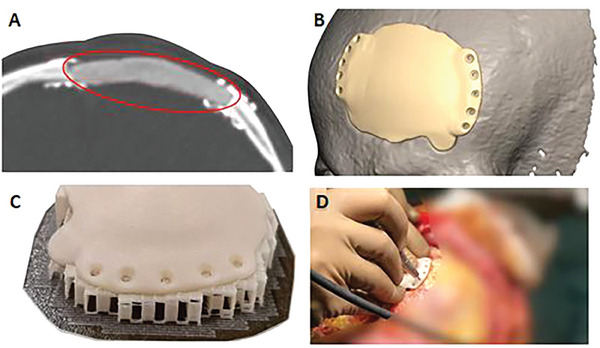
Personalized maxillofacial osteosynthesis system: A) computed tomography image of a frontal lobe defect temporarily filled with neurosurgical cement, B) reconstruction of the patient's skull and the creation of a three‐dimensional implant model fitted to the defective bone, C) 3D‐printed implant manufactured of poly(l‐lactide‐*co*‐d,l‐lactide) and nanohydroxyapatite formulation, and D) surgical procedure of a scaffold implantation in the patient's skull. Reprinted from [www.cyberbone.eu].

This approach is the most promising since it ensures an individualized approach to each patient's needs, in terms of the shape and the chemical composition of the implant. Although this process is time‐bound and multi‐step, it is certainly worth the effort since it provides the most comprehensive solutions for the patients (**Figure** **5**). For all customized products, the first integral step is to develop an imitation (model) of an anatomically defective tissue that is to be regenerated. These structures are computed from standard medical imaging techniques, such as computed tomography (CT) or magnetic resonance imaging (MRI). These methods enable the construction of 3D model of the patient's defective tissues.^[^ ^175^, ^176^ ^]^ Modeling precision is highly dependent on the parameters selected upon imaging with the most important ones, such as the resolution and thickness of the individual layer. This model serves as a substrate for the development of a bioresorbable implant geometric shape. This process is also computer‐aided, often with the use of the haptic systems.^[^ ^177^ ^]^ This model is later used to produce an implant via the typical processing methods, most often, via additive manufacturing. A description of a step‐by‐step procedure for three medical cases aiming to reconstruct craniofacial defects is detailed in the work of Targońska et al.^[^ ^178^ ^]^

This solution is very convenient from certain points of view. First, before the surgery even begins, the trial of physical models of anatomical structures as well as the implant can be manufactured and assessed by the doctor to aid in the planning procedure. In addition, the application of shape‐adapted implant, massively reduces the operational time, in comparison with similar procedures, conducted by using the golden standard methods, such as the auto‐ or the allografting, even by 50–70%. This ensures significant work convenience and increases the patient's safety. This approach also allows to produce several implant copies, in case of the occurrence of damage, the doctor may continue with a new piece. In conclusion, in the case of polymeric biomaterials and unlike titanium protheses, it is possible to adjust their shapes, if needed, in the operating room by using the standard surgical tooling, e.g., to drill holes for fixations, level the surface, etc.

## 3 Applications of Resorbable Biomaterials

Resorbable biopolymers are used in many fields of medicine, from the standard outpatient procedures, e.g., removal of birthmarks, through digestive tract operations, intradermal suturing, organs’ ligation, ophthalmological procedures (e.g., strabismus surgery), microsurgeries (e.g., peripheral nerve anastomosis), to the complex techniques involving bone substitutes. Surgical sutures are currently the most often used standard absorbable medical devices with the first utilized device, being the plain catgut, i.e., collagen fibers obtained from healthy sheep or cattle undergoing enzymatic digestive degradation in the human body.^[^ ^179^ ^]^ Nowadays, surgical sutures are made of synthetic materials, such as PLGA, glycolide, *ε*‐caprolactone, poly‐4‐hydroxybutyrate, or poly(p‐dioxanone) that are absorbed via the hydrolysis process. These threads are available in various thicknesses, ranging from 0.01 to >1.10 mm, and different types, i.e., mono‐ and multifilament. Depending on the biopolymer type, they also differ in handiness, resorption time (max. 180 days), and tissue support profile that can be adjusted to a specific type of sutured wound/tissue or patient's age. Work comfort that can be provided by a vast array of suture materials and suturing methods, results from the observations and the medical procedures that have been performed over many decades.^[^ ^180^, ^181^, ^182^ ^]^ Surgical meshes, loosely woven sheets for temporary supports are another commonly used absorbable medical device that are often utilized to treat wounds without stretching their edges. This type of prosthesis is mainly applied for the treatment of post‐operative and post‐traumatic wounds, hernias, or reparation of abdominal wall defects.^[^ ^183^ ^]^ Day et al. used the PGA mesh with 45S5 Bioglass, which proved in vivo increased vascularization, thereby ensuring a good supply of nutrients and oxygen to new tissue,^[^ ^184^ ^]^ while the more recent work by Perez‐Amodio et al. used PLA‐glass fibrous mats^[^ ^185^ ^]^ and showed increased vascularization, granulation tissue formation, collagen deposition, and accelerated wound closure in vivo. However, the most extensively used meshes are only partially resorbable and are made of half of glycolide copolymer fibers and the other half is composed of non‐resorbable polypropylene yarns. The resorbable part, which can fully be absorbed within 70 days, provides stiffness and improves convenience during the application, while the non‐absorbable mesh part remains in the body, creating a scaffold for the collagen structure to overgrow.^[^ ^186^, ^187^ ^]^ The last class of the required materials are the implantable surgical sheets, i.e., films made of carboxy‐methyl‐cellulose and hyaluronic acid, oxidized and regenerated cellulose, poly(*p*‐dioxanone), copolymers of lactide or *ε*‐caprolactone. They are recommended for adhesion, ossification prophylaxis, and wound dressing.^[^ ^188^ ^]^ Despite these many promising applications, to date, only a few composite systems for soft tissue engineering have reached clinical use in humans. However, it is envisaged that these materials and their biological performance will continue to improve, and society at large will soon experience increasing clinical studies and applications in these areas.

### 3.1 Dental Tissue Engineering

Teeth are a delicate dental organ and they play a pivotal role in the daily digestion of food and speech. The teeth and the periodontal tissues that support the teeth are constantly confronted with a harsh oral environment, which leads to the inflammation and damage of the dental structures, including pulp, dentin, periodontal ligament (PDL), cementum, and alveolar bone. Indeed, tooth decay and periodontal diseases are two of the most common chronic diseases.^[^ ^189^, ^190^ ^]^ More than 90% of all the population over the age of 20 in the United States, have some degree of tooth decay.^[^ ^189^ ^]^ In addition, it was estimated that 47% of the population in the U.S.A., aged 30 years or older, suffered from periodontal diseases.^[^ ^190^ ^]^

Biomaterial‐based tissue regeneration is a promising strategy to replace damaged dental and supporting structures and to restore their biological functions. Biomaterials play pivotal roles during tissue regeneration, including acting as cell carriers, providing mechanical support, and controlling the delivery of bioactive molecules. As the biomaterials serve as temporary templates for tissue regeneration, they should be biocompatible and biodegradable. Many types of biodegradable materials have been explored for dental tissue regeneration. **Table** **1** summarizes the biomaterials that are commonly tested for dental tissue regeneration.

**Table 1:** Summary of the commonly used biomaterials for dental tissue regeneration.

Biomaterials	Target tissues	Characteristics	References
Collagen	Pulp, Periodontal ligament (PDL)	Major ECM component, excellent biocompatibility, low mechanical properties, need to be combined with other biomaterials to enhance mechanical properties.	[191, 192, 193, 194]
Gelatin	Pulp, dentin, periodontal tissues	Hydrolysis product of collagen, water‐soluble, easily modified to incorporate other bioactive molecules, low mechanical strengths.	[195, 196, 197, 198]
Chitosan	Pulp, alveolar bone, PDL	Good biocompatibility, antibacterial activity, need to be combined with other biomaterials to enhance properties.	[199, 200, 201, 202]
Alginate	Pulp, alveolar bone	Ionic cross‐linking, ease of manipulation, low cell adhesion, poor dimensional stability	[203, 204]
Hyaluronic acid	Pulp, PDL	Excellent biocompatibility, regulating osmotic pressure and tissue lubrication, promoting healing, rapid degradation	[205, 206, 207]
Fibrin	Pulp, dentin	Excellent biocompatibility, activation by thrombin to assemble into fibrin gels, binding sites for growth factors and integrins, rapid degradation	[208, 209, 210]
PLA, PLGA	Pulp, dentin, alveolar bone PDL	Controlled degradation rate, tunable mechanical strengths, good biocompatibility, lack of cell recognition sites.	[211, 212, 213]
PCL	Alveolar bone, PDL	Controlled degradation rate, tunable mechanical strengths, good biocompatibility, slow degradation rate, lack of cell recognition sites.	[214, 215, 216, 217]
Hydroxyapatite (HA)	Alveolar bone, cementum	Similar chemical composition to bone, osteoinductive, direct bonding effect to natural bone, slow degradation rate.	[218, 219, 220, 221]
Beta‐tricalcium phosphate (*β*‐TCP)	Alveolar bone, cementum	Similar chemical composition to bone, bioabsorbable, osteoinductive, fast degradation rate.	[222, 223]
Biphasic calcium phosphate (BCP)	Alveolar bone, cementum	Mixture of HA and TCP, tunable degradation rate, osteoinductive.	[224, 225, 226]
Bioactive glass (BG)	Alveolar bone, cementum	Osteogenic and angiogenic activities, tunable degradation rate, low fracture resistance	[227, 228]

Since each biomaterial has certain and peculiar disadvantages, composite biomaterials that combine the advantages of different components, are increasingly, being used for dental tissue regeneration.^[^ ^229^, ^230^ ^]^ Composite biomaterials can better mimic the compositions of natural ECM to support cell growth and tissue formation. The ECM of periodontium is composed of soft and hard tissues, therefore, polymers and mineralized inorganic biomaterials are often combined as templates to support periodontal tissue formation.^[^ ^231^ ^]^ However, most biomaterials that serve as scaffolds cannot provide the biological signals necessary to regulate cell proliferation, chemotaxis, differentiation, and tissue formation. Therefore, bioactive molecules are widely incorporated into the biomaterials to control cell fates and guide tissue regeneration.^[^ ^232^ ^]^ Most of the bioactive molecules^[^ ^233^ ^]^ contain growth factors, such as vascular endothelial growth factor (VEGF), platelet‐derived growth factor (PDGF), bone morphogenetic proteins (BMPs), insulin‐like growth factor (IGF), and transforming growth factor beta (TGF‐*β*). In addition, the mixture of bioactive molecules, including platelet‐rich plasma (PRP), platelet‐rich fibrin (PRF), and enamel matrix proteins, have been used to enhance the dental tissue regeneration.^[^ ^234^, ^235^, ^236^ ^]^ The major challenge to achieving desired outcomes is the delivery of bioactive molecules in a controlled manner, to the defective area.

Self‐assembled peptides are a type of new biomaterials that form an ECM‐like architecture and have recently been explored for dental regeneration.^[^ ^237^, ^238^, ^239^ ^]^ These multi‐domain peptides are amphiphilic and are self‐assembled into hydrogels under mild conditions. The addition of oppositely charged ions or change of pH values initiates the physical cross‐linking, and forms nanofibrous peptide hydrogels. For example, RADA16 peptide is assembled into nanofibers in a neutral pH solution to form a three‐dimensional (3D) hydrogel.^[^ ^238^ ^]^ When compared to conventional biomaterials, these self‐assembled peptides have custom‐designed molecular compositions and configurations and they can be further modified by other functional motifs to obtain better biological properties. However, peptide biomaterials usually have very low mechanical strength that limits their applications in regenerative dentistry.

When biomaterials are used for dental regeneration, they are fabricated into 3D scaffolds with high porosity and adequate mechanical stability to support cell growth. The pores within the scaffold must be interconnected to facilitate cell migration. The degradation rate of the scaffold must be tailored to match the rate of new tissue formation. In addition, the scaffold should have suitable surface recognition able to facilitate cell‐material interactions. Depending on when the scaffolds are shaped, they can be classified as pre‐formed scaffolds and injectable scaffolds. A pre‐formed scaffold has a definite shape and size, prior to the implantation, while an injectable scaffold, forms the shape after injecting it into the defective area. An injectable scaffold holds several advantages, including, the performance in a minimally invasive manner to reduce the risk of infection and improve comfort, readily fixing any irregularly shaped defects, and facilitating cell adhesion and growth, over a pre‐formed scaffold. Given the complicated morphology and structures of dental tissues, injectable scaffolds are more attractive than the pre‐formed scaffolds.

#### 3.1.1 Pulp Regeneration

The initial scaffolds that were used for the pulp regeneration were pre‐formed scaffolds, and most of the scaffolding materials were PLA or collagen sponges.^[^ ^240^, ^241^, ^242^ ^]^ These studies showed that the dental pulp stem cells differentiated and formed pulp‐like tissues under conducive conditions. However, the tooth root canal has an irregular shape with only a small opening at the apical end; therefore, injectable scaffolds are preferred for pulp regeneration. When collagen was injected inside the root canal and transplanted into a nude mice, pulp‐like vascular tissue was regenerated in the canal space after a few weeks.^[^ ^243^ ^]^ However, the low mechanical property of the collagen gel led to the contraction of the construct. Restylane, a HA‐based gel, approved by the FDA for certain applications, was tested as an injectable hydrogel for dental pulp regeneration.^[^ ^205^ ^]^ An in vitro study showed that the restylane gel supported the viability of stem cells of the apical papilla and their odontoblastic differentiation. However, it is unknown whether this material promotes in vivo pulp tissue regeneration. PuraMatrix a commercial peptide hydrogel, was tested for pulp regeneration^[^ ^244^ ^]^ which supported the DPSC survival, migration, and capillary network formation without adding exogenous growth factors. Similarly, the self‐assembling peptide amphiphiles were also evaluated as injectable cell carriers for pulp regeneration purposes.^[^ ^237^ ^]^ These studies proved the feasibility of regenerating pulp‐like tissues when combining dental stem cells with injectable biomaterials.

**Figure 6:**
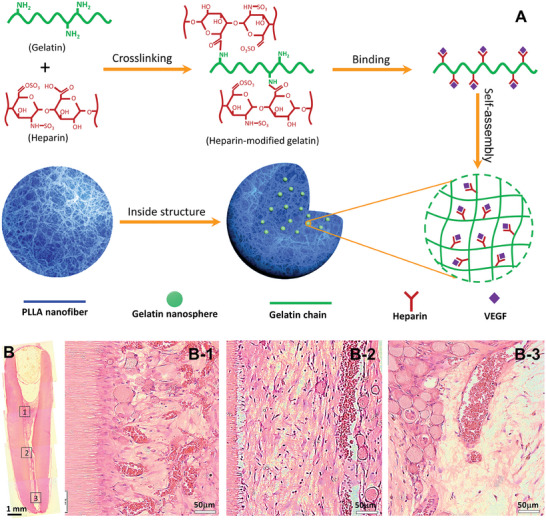
A) Schematic illustration of the synthesis of heparin‐conjugated gelatin nanospheres and the hierarchical VEGF‐loaded nanofibrous microspheres and B) H&E stained images of regenerated pulp‐like tissues in the full‐length root canal after in vivo implantation for nine weeks. Adapted from.^[^ ^247^ ^]^ Copyright 2016 Elsevier Ltd.

The human tooth root has a long canal with a narrow opening that limits nutrient diffusion and new blood vessels in‐growth. Therefore, fast revascularization has long been a challenge for pulp regeneration in a full‐length root. In one study, angiogenic growth factor VEGF was incorporated into injectable RGD‐alginate/laponite hydrogel microspheres for endodontic regeneration.^[^ ^203^ ^]^ The subcutaneous implantation experiment indicated the fact that the VEGF‐loaded microspheres significantly enhanced the pulp‐like tissue regeneration and new blood vessel formation. Further examination, however, showed that the regenerated vascularized soft tissue had a length of <4 mm, which was considerably shorter than the length of a full‐length root, i.e., between 11–13 mm. Similar results were reported when DPSCs were co‐cultured with umbilical vein endothelial cells (HUVECs) to enhance angiogenesis.^[^ ^245^ ^]^ Our group,^[^ ^246^ ^]^ recently developed a unique hierarchical nanofibrous microsphere system for a full‐length pulp regeneration.^[^ ^246^ ^]^ In this system, VEGF was encapsulated into heparin‐conjugated gelatin nanospheres and the VEGF‐loaded nanospheres were further immobilized into nanofibrous PLLA microspheres (**Figure** **6**). Since VEGF has binding domains with heparin, the binding of VEGF to heparin‐conjugated nanospheres protected the VEGF from enzymatic degradation and prolonged its sustained release. Indeed, the release of VEGF from the hierarchical spheres was regulated by a multiple‐layer manner, including the binding with the heparin, the degradation of heparin‐conjugated nanosphere, and the physical adsorption of the microsphere nanofibers. This hierarchical injectable microsphere scaffolding system provided an excellent environment for the pulp tissue regeneration in a full‐length tooth root.

The in vitro study showed that the nanofibrous microspheres integrated the ECM‐mimicking architecture with a highly porous injectable form and efficiently supported the DPSC growth and pulp‐like tissue formation. The in vivo study further indicated the successful regeneration of highly vascularized pulp tissues that filled the entire two thirds of the root space and reached the coronal third space of the canal. The result was further confirmed by using a multi‐functional peptide‐conjugated non‐viral gene nanocarrier,^[^ ^198^ ^]^ indicating the feasibility of regenerating to pulp tissue in a full‐length root with one end sealed by using a single installation, which is a significant step towards regenerative endodontics in clinical settings.

#### 3.1.2 Dentin regeneration

**Figure 7:**
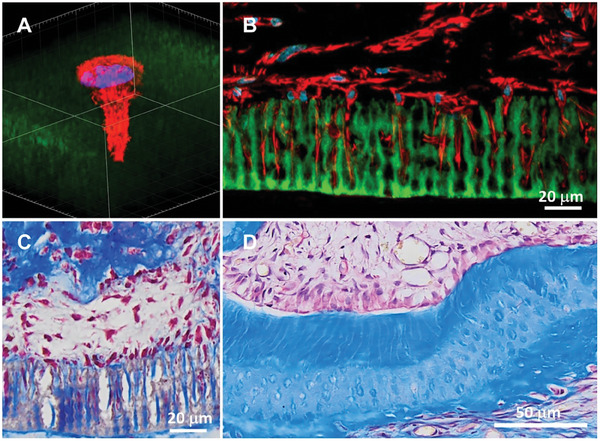
Nanofibrous tubular matrices‐guided tubular dentin regeneration. A) A confocal image showing a DPSC extending its process into a tubule, B) Confocal image after the DPSC/tubular matrix construct was cultured in vitro for seven days, C) Trichrome staining after the DPSC/tubular matrix construct was cultured in vitro for two weeks and D) Tubular dentin regeneration after implantation for four weeks. Adapted from ref.[252] Copyright 2017 WILEY‐VCH Verlag GmbH & Co. KGaA, Weinheim.

Dentin is composed of millions of well‐organized dentinal tubules^[^ ^248^ ^]^ that have diameters of between 2–4 µm. This tubular architecture provides channels for fluid/stimulus signals transformation and mechanical support for the tooth. Therefore, regeneration of the tubular dentin is essential to fully recover the physiological function of a tooth. Due to the technical challenge of fabricating a tubular matrix to guide the DPSC migration and differentiation, the effort of regenerating tubular dentin was not successful and only the bone‐like mineralized tissues or the sporadic dentinal tubules were formed in the root canal chamber after an in vivo tissue regeneration.^[^ ^212^, ^249^ ^]^ For example, DPSCs formed a dentin‐like structure in a small area of the HA/TCP microparticle surfaces, 6 weeks post‐transplantation.^[^ ^250^ ^]^ When the dental progenitor cells were seeded onto the PLGA scaffolds and inserted into a human root fragment, a layer of mineralized tissue was deposited on the root canal wall, four months after implantation.^[^ ^212^ ^]^ However, the well‐organized tubular dentin structure was not observed. A maskless micropatterning technology was recently developed to create a 3D nanofibrous tubular matrix for tubular dentin regeneration.^[^ ^251^ ^]^ Firstly, the micropattern was designed by using computer software. Subsequently, a laser micro‐dissection machine was applied to the nanofibrous matrix to generate tubular matrix by precisely controlling the fabrication parameters, e.g., laser output power, aperture, laser writing and laser pulse frequency. The synthetic tubular matrix had the same size and density of the tubules when compared to those of the natural dentin. When the DPSCs were cultured on the tubular matrix, in differentiation media for 2 weeks, a layer of odontoblasts with long processes was aligned on the tubular matrix surface, confirming the essential role of the tubular architecture to guide the DPSC polarization and differentiation (**Figure** **7**). The in vivo implantation experiment further showed that a layer of a well‐ordered tubular dentin with an average length of ≈45 µm was deposited on the tubular matrix. This work demonstrated the fact that the tubular architecture of the matrix was a pivotal physical factor for tubular dentin regeneration. Future work should integrate this micropatterning technique with other scaffolding fabrication methods and generate a hierarchical matrix for pulp/dentin regeneration.

#### 3.1.3 Alveolar bone regeneration

For several decades, Guided Bone Regeneration (GBR) has been used for alveolar bone regeneration in clinical settings.^[^ ^253^ ^]^ Polymeric materials are usually selected as the GBR barrier membranes that aim to prevent soft tissue downgrowth and maintain the defect space during alveolar bone regeneration. The initial GBR membranes, e.g., expanded polytetrafluoroethylene (ePTFE), were non‐resorbable. Consequently, a second surgery is needed to retrieve the ePTFE after new tissue formation which increases the risks of infection and site morbidity. Therefore, the current research focuses on the development of resorbable GBR membranes for alveolar bone regeneration.

Collagen has been extensively explored as a GBR resorbable membrane and there have been several collagen‐based commercial GBR membranes in the market.^[^ ^253^ ^]^ Collagen membranes support osteogenic differentiation, however, their low mechanical strength impairs the function of space maintenance for new bone ingrowth. To increase the mechanical strength, synthetic biodegradable polymers, especially poly(alpha‐hydroxy esters), have been tested as resorbable GBR membranes. However, these synthetic biomaterials often need surface modification or their combinations with other bioactive components to promote cell adhesion, migration, and differentiation.

A composite hydrogel, composed of hydroxyapatite‐calcium sulfate‐hyaluronic acid (HA/CS/HRA) with the encapsulation of collagenase was developed to repair alveolar bone defects.^[^ ^254^ ^]^ The combination of the three components, provided a fast absorption of the substrates that facilitated new bone formation. In addition, the release of collagenase from the composite hydrogel could initiate bone remodeling by accelerating wounded trabecular bone digestion. Twelve weeks after implantation, histological images showed that ≈78.3% of the defective site was occupied by the newly formed bone in the HA/CS/HRA group. However, a rapid initial burst release (>80% within the first 24 h) of collagenase from the hydrogel was observed, indicating that an improvement of the collagenase release is needed. In another study, metformin was used as an anti‐inflammatory agent and it was encapsulated into a *β*‐TCP/chitosan/mesoporous silica composite hydrogel.^[^ ^255^ ^]^ The metformin‐loaded composite scaffold promoted alveolar bone regeneration in a periodontitis rat model. Other materials/drugs that are often used for antibacterial/anti‐inflammation during alveolar bone regeneration include: Ag and ZnO nanoparticles, aspirin, and tetracyclines.^[^ ^256^ ^]^

Since periodontitis‐induced alveolar bone loss is a chronical inflammation disease. An immunomodulatory strategy was recently developed for alveolar bone regeneration.^[^ ^257^ ^]^ This strategy is targeted to regulate immune cells, especially macrophages that play a central role in inflammation regulation. It is known that M1 macrophages are pro‐inflammatory and M2 macrophages are pro‐healing. Therefore, switching of the macrophage phenotype from M1 to M2 is crucial during the wound‐healing process. In one study, interleukin 4 (IL4) was used to moderate macrophage phenotypes during alveolar bone regeneration.^[^ ^257^ ^]^ IL4 was first incorporated into heparin‐conjugated nanospheres, and the binding of IL4 with heparin in the nanospheres protected the IL4 from degradation and controlled its sustained release. Next, the IL4‐loaded nanospheres were encapsulated into the nanofibrous microspheres. In‐vitro and in vivo studies demonstrated the fact that the immunomodulatory microspheres effectively switched the pro‐inflammatory M1 macrophages into anti‐inflammatory M2 macrophages, and therefore significantly enhanced new bone regeneration. Future studies by using large animals are needed to verify the results in a more clinically related disease model.

#### 3.1.4 Alveolar bone/PDL/cementum complex regeneration

Alveolar bone, PDL and cementum are a structural and functional entity of periodontium; therefore, a functional periodontal tissue regeneration should include all the three components. A tri‐layered composite scaffold was fabricated to regenerate alveolar bone, PDL, and cementum.^[^ ^258^ ^]^ In that design, chitin/PLGA was selected as the substrate materials for all the three layers, while bioactive glass ceramic nanoparticles were added to the cementum and alveolar bone layers. In addition, platelet‐rich plasma, fibroblast growth factor 2, and cementum protein 1, were introduced to the alveolar bone, PDL, and cementum layers, respectively. Histological images showed the formation of a new alveolar bone, fibrous PDL and cementum, three months after their implantation in a rabbit periodontal defect model. In another study, cell sheet was used to replace the cementum layer and a biphasic scaffold was fabricated for periodontal regeneration.^[^ ^259^ ^]^ Cementum‐like and PDL‐like tissues as well as new bone were regenerated after the multilayer scaffold was attached to a dentinal slice, followed by subcutaneous implantation for 8 weeks.

PDL principal fibers and Sharpey's fibers were pivotal to anchor a tooth to the alveolar bone.^[^ ^260^ ^]^ One study developed a 3D printing technique to create micro‐patterned channels for PDL principal fibers formation.^[^ ^261^ ^]^ Results indicated that the micro‐patterned PCL matrix increased collagen fiber alignment along the surface of the grooved pillars. One limitation of this study is that the micro‐patterned scaffold could only guide the PDL fibers to align on the surface of the grooved pillars and there were no Sharpey's fiber insertions into the cementum/bone portions.

A multi‐compartmental scaffold was fabricated to regenerate PDL and alveolar bone by using the Melt electrowriting (MEW) technology.^[^ ^262^ ^]^ Human periodontal ligament fibroblasts and primary human calvarial osteoblasts were seeded onto the PDL and the alveolar regions of the scaffold, respectively. Four weeks after the co‐culture in a differentiation medium, high calcium content in the bone region, moderate calcium content in the transition region, and low calcium content in the PDL region, were observed in the construct. However, the insertion of PDL fibers into the bone compartment of the construct was barely observed, suggesting that no functional PDL was regenerated.

A biphasic scaffold for PDL and alveolar bone regenerations were fabricated by two 3D printing techniques.^[^ ^263^ ^]^ Specifically, the PDL compartment was fabricated by a DLP printer, and the alveolar bone compartment was fabricated by a DIW printer. The DLP printer had a high resolution, needed to provide topographical cues to guide the PDL fiber formation and the DIW printer generated the grid structure to support osteoblast growth. Porcine dental follicle‐derived de‐cellularized ECM bioink was further added into the matrix to provide favorable biochemical cues to improve the viability of the encapsulated dental follicle cells and osteogenic differentiation. An in vivo study showed that the biphasic scaffold promoted the regeneration of periodontal tissues, including the PDL fibers and mineralized the alveolar bone. However, the integration of the PDL module with the alveolar bone module is not clear. However, cementum regeneration was not considered in this study.

Overall, significant progress has been made over the last two decades to develop biodegradable materials and fabrication technologies for dental tissue regeneration. Future research should focus on the development of new bio‐inspired ECM‐like materials that can precisely mimic the in vivo micro‐environment to accelerate the pulp/dentin and periodontal tissue regenerations.

### 3.2 Bone drug delivery systems

Methods and technologies used to transport pharmaceutical compounds in the body to achieve a therapeutic effect are considered as drug delivery systems. They include systems and devices that are designed to control the release, absorption, distribution, and elimination of drugs, with the aim to optimize their efficacies and safety. This field is crucial in terms of enhancing the performance of pharmaceutics, ensuring that they reach the targeted site in the correct dosage, and further improve their efficacies, minimize side effects, and improve the patient outcomes.^[^ ^264^, ^265^ ^]^ When a system is employed for the treatment of bone‐related diseases and conditions, it is classified as a bone drug delivery system.^[^ ^266^ ^]^ As the demand for efficient and minimally invasive therapeutic interventions in orthopedics continues to rise, the integration of resorbable materials into the advanced drug delivery platforms emerges as a pivotal strategy, offering accelerated healing, improved patient outcomes, and a paradigm shift in the bone regenerative medicine field. In the last few decades, multiple studies have investigated the potential of using these materials in bone drug delivery applications, taking advantage of their physicochemical and resorbable properties.^[^ ^267^ ^]^ For instance, resorbable nanoparticles are widely used for controlled drug release in applications, such as cancer therapy and bone diseases (e.g., osteoporosis, osteoarthritis), and infections. Examples include the use of hollow PLGA microspheres to treat OA for the delivery of the anti‐inflammatory drug dexamethasone,^[^ ^268^ ^]^ to load anticancer therapeutics in nano‐composites for the controlled drug release of doxorubicin in natural‐based bone cement.^[^ ^269^ ^]^ Similarly, nano *β*‐tricalcium phosphate (n*β*‐TCP) particles reinforced 3D bilayer collagen (COL) scaffold was further investigated for the delivery of leukocyte platelet‐rich fibrin (L‐PRF) cells and growth factors to initiate bone regeneration.^[^ ^270^ ^]^

Various strategies have been developed to overcome the obstacles to effective drug delivery to the bones, while minimizing the negative side effects. Biomaterials are combined to achieve this goal. These strategies^[^ ^271^ ^]^ include: (1) the use of small‐sized nanoparticles to enhance drug penetration and take advantage of the enhanced permeability and retention (EPR) effect, which is a passive drug targeting mechanism, (2) the use of inorganic nanoparticles, such as TiO_2_‐, gold‐, and SiO_2_‐based nanoparticles, to induce endothelial leakiness, creating micrometer‐sized gaps between the endothelial cells, (3) the use of stimuli‐responsive carriers that release payloads in response to intrinsic or extrinsic stimuli, such as: pH, hypoxia, enzymes, glutathione (GSH), reactive oxygen species (ROS), light, temperature, ultrasound, magnetism, and electricity, and (4) the use of targeting ligands to enable active delivery of drugs to specific tissues, immune cells, or tumor cells through ligand‐receptor interactions. Overall, resorbable biomaterials for bone drug delivery require a multifaceted approach. Calcium‐based materials, natural and synthetic polymers, and nanocomposites, each bring their own unique advantages to tissue engineering and clinical applications. Careful selection of materials and their properties is crucial to achieving successful bone drug delivery strategies. The key properties include biodegradation timelines, with calcium‐based materials, having slower resorption rates than synthetic polymers. Polymer degradation yields sub‐products that can influence the local micro‐environment, therefore, precise modulation of these properties is essential to match the therapeutic window for bone regeneration or drug intervention.

#### 3.2.1 Clinical challenges in bone tissue regeneration requiring controlled drug delivery

When it comes to regenerating bone tissue, controlled drug delivery systems are a crucial solution to address various clinical challenges such as: bone cancer, osseointegration, bone tissue infections, etc. In cases like bone cancer, the challenge is to achieve a drug release system that is localized and sustained to fight tumor progression, while reducing systemic side effects. Since the objective is to create drug delivery platforms that aim at the tumor micro‐environment, while still preserving healthy bone tissue, the key challenges are mainly:

- Drug Resistance and Systemic Adverse Effects: Systemic drug delivery can lead to adverse effects throughout the body. Bone tumors, including bone cancer, often develop resistance to chemotherapy drugs, making treatment less effective.^[^ ^272^ ^]^ This resistance can be due to various factors, such as the presence of cancer stem cells and uncontrolled proliferation.^[^ ^273^ ^]^ Bone‐targeted drug delivery systems aim to minimize systemic adverse effects by concentrating the drugs at the tumor sites and prolonging their circulation times.
- Tumor Microenvironment, Complexity and Heterogeneity: The tumor micro‐environment including factors such as tumor‐associated vasculature and stromal cells, which can create additional barriers to drug delivery. Bone tumors are complex and heterogeneous, which makes them a challenging task to develop effective treatment protocols.^[^ ^272^ ^]^ The presence of various barriers, such as unrevealed aetiology, histological diversification, and genomic instability, further complicates drug delivery to the tumor site.^[^ ^274^ ^]^ These barriers can limit the effectiveness of controlled drug delivery systems.
- Limited Drug Accumulation: Delivering drugs specifically, to the bone tumor site, is crucial for effective treatment. However, the bone micro‐environment and blood clearance can limit the accumulation of drugs in the tumor.^[^ ^272^ ^]^ This can reduce the therapeutic efficacy of the treatment.
- Lack of Biomarkers: Bone tumors, including bone cancer, often lack specific biomarkers, which makes it a challenging procedure to target specifically, the tumor cells.^[^ ^274^ ^]^ This can result in a non‐specific drug delivery circumstance and hence, reduced treatment efficacy.

Addressing these challenges is crucial for improving the treatment of bone cancer. Researchers are exploring various approaches, such as nanocarriers and gene therapy, to overcome these challenges and enhance drug delivery to the tumor sites.^[^ ^275^ ^]^

The field of osseointegration necessitates the precise control over drug release to enhance implant‐bone bonding. The main clinical challenges associated with controlled drug delivery in osseointegration are related to infection and the loss of integration.^[^ ^276^, ^277^, ^278^, ^279^ ^]^ Achieving optimal drug concentrations around the implant site promotes accelerated bone formation, mitigates inflammation or potential infections, and prevents implant loosening. The challenge lies in tailoring the drug release kinetics to match the bone healing process, thereby ensuring successful implant integration. Infection is a common complication of implant surgery, and it can lead to implant failure, if not treated promptly and effectively. The treatment of infections in bone tissue can be a difficult task, since it requires targeted delivery of antibiotics to effectively eliminate microbial growth. The main obstacle is the creation of the drug delivery systems that allow for the sustained release of antibiotics, ensuring that therapeutic levels remain constant at the site of infection, without impairing bone tissue growth. This approach helps to prevent the development of antibiotic‐resistant strains and promotes complete eradication of infection. The two key clinical challenges are summarized, thus:

- Limited Drug Accumulation: Delivering drugs specifically to the site of bone infection is crucial for effective treatment. However, as mentioned previously, the bone micro‐environment and blood clearance can limit the accumulation of drugs in the infected area,^[^ ^280^ ^]^ thereby, reducing the therapeutic efficacy of the treatment.
- Bacterial Resistance and biofilm formation: Bacterial resistance to antibiotics is a growing concern in the treatment of bone infections. Bacteria in bone infections can form biofilms, which can make it difficult for drugs to penetrate and kill the bacteria. This can make it a challenging task to develop effective treatment protocols and reduce the effectiveness of the controlled drug delivery systems. Indeed, there is a research need to develop targeted and efficient drug delivery systems for bone infections.

Local drug delivery systems that carry osseointegration drugs, i.e., drugs that can promote bone growth or angiogenesis, on titanium implants have been shown to have a better effect than systemic administration of drugs to promote osseointegration before and after implantation.^[^ ^277^ ^]^ However, the development of efficient local therapeutic delivery strategies is imperative to avoid systemic toxicity caused by antibiotics that are administered systemically at a high enough dose to reach the necrotic area and eradicate the infection.^[^ ^279^ ^]^ Therefore, it is important to construct the local drug delivery systems on titanium‐based implants to improve osseointegration.^[^ ^278^ ^]^

Efficient and ethical drug testing requires accurate replication of the clinical scenario through functional bone models. However, the creation of in vitro bone tissues for the study of controlled drug delivery is challenging due to the complexity of the bone tissue, difficulty in mimicking the microenvironment, limited drug delivery options, and limited drug efficacy. Bone tissue is a complex and dynamic tissue, and hence, its micro‐environment plays a crucial role in regulating cell behavior and drug delivery. For instance, the highly mineralized nature of bone can affect the drug release kinetics. In addition, the drug delivery system must be biocompatible, biodegradable, and able to target specific cells.^[^ ^281^ ^]^ For example, the group of Scalzone *et. al*.,^[^ ^282^, ^283^ ^]^ established different in vitro models to study the tissue micro‐environments and bone disease,^[^ ^284^ ^]^ which can be exploited in drug screening to reduce and refine the use of animals. It is to be considered that even if a drug delivery system is designed successfully, the efficacy of the drug may be limited due to factors, such as poor bioavailability, rapid clearance, and off‐target effects.^[^ ^273^ ^]^ Therefore, models must be developed to overcome these challenges when assessing drugs in vitro.

#### 3.2.2 Innovative approaches for controlled drug delivery

In the field of bone drug delivery, there are innovative approaches that are changing the pathway that therapeutic interventions are done. These methods offer precise, localized, and sustained drug release, which can improve the treatment efficacy and safety for different bone‐related conditions. These innovative approaches, address the challenges, such as: achieving sustained drug concentrations at the target site, minimizing systemic side effects, and tailoring drug release to match the specific needs of bone regeneration or disease treatment. However, challenges such as scalability, biocompatibility, and regulatory considerations, still need to be addressed. Robust testing and optimization are crucial to ensure the safety and efficacy of these approaches. Moreover, the integration of these innovations with patient‐specific factors, such as bone anatomy and pathology, will contribute to personalized and patient‐centric treatment strategies. Therefore, these innovative methodologies are revolutionizing the field of controlled bone drug delivery by having the potential to address the associated clinical challenges and open new avenues for the precise and effective therapeutic interventions in bone‐related conditions.

##### Drug encapsulation: bead/micro and nanoparticles

One route to achieve controlled release profiles is through drug encapsulation by using biocompatible carriers, e.g., microparticles or nanoparticles. This protects the drug from degrading prematurely, improves bioavailability, and provides modulation of release kinetics. Nanoparticles, because of their size and surface properties, offer enhanced cellular uptake and targeted drug delivery. Synthetic polymers, such as biodegradable PLGA and PLA, have been widely exploited to encapsulate drugs because of their stability, and hence, to increase the circulation time and the bioavailability of the drug in chronic diseases and/or conditions that require sustained drug delivery.^[^ ^285^ ^]^ In addition, versatile and biodegradable polymers, such as poly(ethylene glycol) PEG have been used in the development of drug carrier and related advanced systems. For instance, advanced block copolymer prodrug‐based polymersome nanoreactor, has been designed to facilitate oxidation/chemotherapy via specific activation at tumor sites. The tumor acidity‐triggered protonation, activates the pro‐drugs by generating H_2_O_2_, release the active camptothecin drugs at the site, and hence, synergistically killing cancer cells and suppressing tumor growth.^[^ ^286^ ^]^ Multifunctional systems that combine therapeutic agents with imaging agents or targeting ligands, have been designed by engineering these carriers, which can augment their therapeutic potentials. Materials‐based carbon quantum dots, graphitic carbon dots and other analogues, have attracted significant attention due to their propensity to be synthesized at nano sizes, ability to be modified to contain several varying functional groups to incorporate into ligands, contrasting agent and graft drugs, among other advantages.^[^ ^287^ ^]^

##### Layer‐by‐layer approaches

Another approach is the layer‐by‐layer (LbL) assembly, which involves the deposition of alternating layers of oppositely charged materials onto a substrate. This method enables the precise control over drug release kinetics by adjusting the layer thickness and composition. It also facilitates the incorporation of various therapeutic agents, biomolecules, and growth factors, thereby, promoting specific cellular responses. This approach is particularly useful in bone tissue engineering,^[^ ^288^ ^]^ where the sequential release of growth factors can mimic the natural healing cascade. This technique has been used in different systems and approaches, such as polymeric fibrous membranes and scaffolds, implants^[^ ^289^ ^]^ for the sustained release of growth factors,^[^ ^290^, ^291^ ^]^ antibacterial and anti‐inflammatory agents.^[^ ^292^, ^293^ ^]^

##### Additive manufacturing and microfluidics

The additive manufacturing or the 3D printing method, offers the ability to create intricate scaffolds with controlled geometries and internal architectures. By embedding drugs within the scaffold material, additive manufacturing enables a localized drug delivery, directly from the implant. For instance, biodegradable polymers can be exploited into 3D structures for controlled drug release, such as in the case of the 3D printed polycaprolactone (PCL) scaffolds for the purpose of surmounting the heat lability of rifampicin, an antibiotic used in osteomyelitis treatment.^[^ ^294^ ^]^ Similarly, 3D printed calcium phosphate cement scaffold, coated with anti‐cancer drugs, was explored as an approach to treating bone cancer; in this case, 5‐fluorouracil (5‐FU) was used as a drug model to inhibit the growth of osteosarcoma.^[^ ^295^ ^]^ Meanwhile, microfluidics allows for the precise manipulation of the fluids at the micro‐scale level. This technology facilitates the formation of drug‐loaded microspheres or microparticles with tune‐able properties, hence enabling customized drug release profiles. Some of the commonly used polymers for the manufacturing of these synthetic hydrogel microspheres by exploiting microfluidics comprise of water‐soluble polymers, such as: polyethylene glycol, alginate, hyaluronic acid, and gelatin and gelatin‐methyl acrylic polymers, among others. These microspheres (between 45 to 1680 µm in diameter) are excellent carriers of various drugs, such as: cytokines, antibiotics growth factors, etc., as summarized elsewhere.^[^ ^296^ ^]^

### 3.3 Regenerative Surgery

#### 3.3.1 Maxillofacial, Craniofacial, and Orthopedics

**Figure 8:**
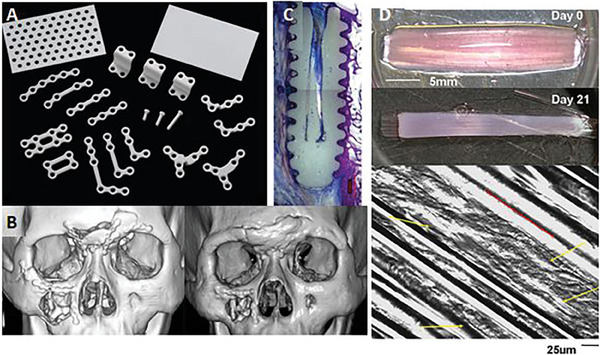
Applications of bioresorbable composites: A) OSTEOTRANS MX® implants for cranial, oral, and maxillofacial use. Reproduced from ref.[303] under the CC‐BY 4.0 license, B) OSTEOTRANS MX® implants used for facial fractures, immediately postoperative (left) and 2 years postoperative (right), showing implant resorption and some excess frontal bone growth. Reproduced with permission from ref. [300] C) Biosteon HA/PLLA interference screw 12 months post‐implantation, showing a new bone growth in a sheep model. Reproduced with permission from^[^ ^304^ ^]^ and D) Glass fiber reinforced collagen scaffolds for muscle tissue engineering, showing cell orientation (red arrow) along glass fibers. Reproduced with permission from ref.[305]

***Maxillofacial surgery*** includes facial trauma surgery for treatment of various types of fractures and bone injuries, resulting from traffic or construction accidents, fights or sport injuries. Most often, fractures are related to mandible, maxilla, or eye sockets bones. Another reason for surgical intervention in the craniofacial area is the malocclusions, associated with abnormal development of the facial bones. This usually applies to such anatomical defects as excessive protrusion of the mandible (progenia), narrowing of the jaw, abnormalities in the structure of the chin or the so‐called “open bite”. To enable patients that are affected by this type of disease to function properly or fully recover, complex surgical procedures are performed, with the main purpose, being an internal osteosynthesis, i.e., bone fracture fixation with connecting elements. The golden standard for rigid fixation in maxillofacial surgeries, e.g., orthognathic operations, maxillofacial fractures, and reconstructive surgery, are the conventional titanium plates and screws for osteofixation that provide uninterrupted bone healing and optimal remodeling. However, these standardized products are non‐resorbable.^[^ ^297^ ^]^ Therefore, the market started to offer resorbable fixation systems, such as: meshes, foils, screws, and plates, which are intended for fracture repair and maxillofacial reconstruction. As reported by Laine et al.,^[^ ^297^ ^]^ bioresorbable devices are safe to use in orthognathic procedures, and are particularly interesting for the treatment of pediatric craniofacial bone fractures with bioresorbable plates since they eliminate the need for a second surgery. In fact, bioresorbable plates do not provide the same fracture fixation stiffness as those made of titanium alloy, since the support is sufficient in the case of young patients. In addition, they enable further mandible growth, tooth eruption and the muscular forces that influence satisfactory bone modeling.^[^ ^298^ ^]^ Zanetti et al. used the flexible PCL/HA composite sheets to reconstruct skull base defects in seven patients, with a satisfactory outcome after a minimum of an 18 month follow‐up.^[^ ^299^ ^]^ In addition, a PLLA/HA composite has been marketed as OSTEOTRANS MX for the repair of maxillofacial fractures (**Figure** **8**A,B). This material has been used in several clinical studies,^[^ ^300^, ^301^, ^302^ ^]^ with group sizes ranging from 17–35 patients, with most cases showing satisfactory outcomes, including bone growth into the material and absorption of the implant, although a small number of patients experienced a foreign body reaction.

The ***craniofacial surgery*** primarily deals with the skull bone defects, resulting for example, from cancer or mechanical injuries. Bone flap removal in neurosurgical procedures is widely accepted to deal with those difficult clinical situations. However, the natural head shape and the brain protection against mechanical trauma, need to be truly assured.^[^ ^306^, ^307^, ^308^ ^]^ The first‐choice approach in the case of cranioplasties is auto‐ or allografting. If the defect is too large, the dimensionally stable and durable implants made of titanium alloys or biostable thermoplastic polymers, i.e., polyetheretherketone, are used. Since the presence of a foreign object in the body may result in complications, e.g., possible rejection or inflammation, bioresorbable alternatives are constantly sought. The literature proposes innovative absorbable materials for the correction of, e.g., calvarial defects. Chatzipetros et al.^[^ ^309^ ^]^ conducted an in vivo test on animals with a hydrogel scaffold being a combination of chitosan and nanohydroxyapatite. The complex was proved to ensure a statistically significant increase on the surface of the newly formed bone in the rats’ lateral area, inward of the middle sagittal seam. On this basis, it was concluded that this scaffold should be considered as a suitable biomaterial for a guided bone regeneration. Another route to treat this defect was studied in vivo by Yun et al.^[^ ^310^, ^311^ ^]^ A 3D printing method was proposed to produce scaffolds from PLA with and without HA. Microcomputed tomography, histologic and histomorphometric analyses, proved that the scaffolds were biocompatible and they integrated well with the bone defect margin to create proper space for new bone formation. Another approach comprising of nanohydroxyapatite and polylactide‐glycolide microspheres was tested on animals for the rats’ skull bone defects. The composition was compared with the commonly used xenograft (Bio‐Oss). The naked eye observations revealed clear boundaries of the skull defects and new bone formation.^[^ ^312^, ^313^ ^]^ It is noteworthy that all the above‐mentioned cases concern tests that were carried out on animals; while, the first clinical applications of bioresorbable implants for maxillo‐ and craniofacial reconstructions on human patients were presented by Targońska. et al.^[^ ^178^ ^]^

***Orthopedic surgery*** focuses on diagnostics, surgical and conservative treatments of the musculoskeletal system diseases, particularly the bone skeleton. The bioresorbable products for temporary tissue fixation, include suture anchors/tacks for soft tissue to bone fixation, interference screws for ligament repair, meniscal repair tacks, fracture fixation screws, pins and plates, e.g., for ankle, tibia or fibula fracture treatment.^[^ ^314^, ^315^ ^]^ Clinically used orthopedic polymer‐based implants, usually comprise of PLA, poly‐l/dl‐lactide, poly‐l‐lactic or PLGA modified with HA or TCP, e.g., Biosteon (Figure 8C,D) and Biolok (Biocomposites Ltd./Stryker) or Milagro screw made of BIOCRYL RAPIDE material (DePuy Synthes). Bio‐resorption of these screws and their osteoconductivity have been confirmed from clinical trials in anterior cruciate ligament reconstruction surgeries.^[^ ^304^, ^316^, ^317^ ^]^ An interesting bioresorbable stent for the anterior cruciate ligament reconstruction was proposed by Ficek et al.^[^ ^318^ ^]^ The PLA stent was tested in vivo (on a sheep model) and it exhibited a reduction of the tunnelling of tibial and femoral diameters and volumes. In addition, the microstructural parameters of the bone adjacent to the tunnels, tended to be better in the PLA stent when compared to the autografted control group.

#### 3.3.2 Cardiac surgery/Cardiology

In the case of this branch of medicine, absorbable stents and heart valves are of particular interest. A stent is a type of cylindrically shaped coil with a thin meshed structure that serves as an endovascular prosthesis to maintain the lumen of dilated blood vessels. They were developed to overcome acute complications of plain balloon angioplasty (major flow‐limiting dissection, acute thrombosis, and vessel recoil). Later, they became the cornerstone of percutaneous coronary intervention (PCI) because of a reduced restenosis rate and clinical events in follow‐up. There are three types of stents used in medical practice: metal (BMS‐Bare Metal Stent), drug‐coated (DES‐Drug Eluting Stent) and resorbable (BVS‐Bioresorbable Vascular Stents). After the assimilation of the drug‐eluting stents as care standard, due to the limited short‐term restenosis, bioresorbable scaffolds were developed to avoid the metallic caging of the vessels. Several theoretical advantages, such as, the removal of the metallic foreign body to reduce inflammation and neo‐atherosclerosis, hence restoring the vasomotor function, or allowing for future grafting or the unlimited repeats of revascularization of the vessel arose. The main advantage of bioresorbable products is that they provide scaffolding to the vessels in the early healing phase post‐surgery and later on, when their role is completed, they undergo biodegradation by releasing the vessel from scaffolding.^[^ ^319^ ^]^ The poly‐l‐lactic acid–based Absorb scaffold (Abbot Vascular, USA) was the first to be widely marketed for in‐human, multicentre, clinical trials. However, the studies proved that, when compared to the market‐leading metal stents, the use of Absorb BVS led to a higher risk of stent thrombosis. After the discontinuation of Absorb BVS trials, other platforms have gained some interest. Among them, the magnesium bioresorbable stent (Magmaris, Biotronik) stood out, but some others are currently, undergoing development or in their early stages of clinical testing.^[^ ^320^, ^321^, ^322^ ^]^ Efforts are also made to introduce novel materials,^[^ ^323^, ^324^ ^]^ such as desaminotyrosine polycarbonate polymer, salicylates, *ε*‐caprolactone and poly(d,l‐lactide‐coglycolide) for these purposes.

The highest interest in advanced heart valve tissue engineering is to provide solutions for the increasing number of patients that require valve replacements. Various biomaterials, whether biological, synthetic, or a combination of both, can be used to create scaffolds that may promote host tissue regeneration after implantation. Frequently, the implantation procedure starts with the harvesting of cells from the patient, which are further expanded onto the scaffold's surface. These scaffolds are usually offered in the form of durable shapes or hydrogels. Porous scaffolds can be produced via 3D printing from various polymers, i.e., PGA, PLA, or collagen. Lueders et al.^[^ ^176^ ^]^ detailed the fabrication of custom‐made human heart valve scaffolds via a selective laser‐sintering 3D printing for subsequent seeding with vascular cells from human umbilical cords. The research focused mainly on suitable scaffold material that allowed the implementation of both, the printing as a production method and the cell‐seeding process, while meeting all the above‐mentioned requirements. Hydrogel scaffolds are formed by the crosslinking of hydrophilic polymers through various reactions, e.g., free radical polymerization. Hydrogels are advantageous because they have high water contents, which allow the ease of penetration with nutrients.^[^ ^325^ ^]^ Another interesting approach was proposed by Sell et al.^[^ ^326^ ^]^ who suggested the electrospinning of biopolymers for cardiovascular tissue regeneration. It was assumed that such vascular prostheses could be produced from biocomponents, such as collagen, elastin, gelatine, fibrinogen, or silk fibroin alone or in combination with resorbable synthetic polymers.

#### 3.3.3 Aesthetic medicine and plastic surgery

Aesthetic medicine deals with ensuring high life quality of healthy people through preventive actions, largely focusing on the limiting of skin aging or renovation processes. Plastic surgeries, on the other hand, handle the reconstructions of congenital and acquired body defects as well as the corrections of the defective body appearance of real or tangible nature.^[^ ^327^ ^]^ In both cases, substances, i.e., the so‐called fillers, are commonly used with the main aim of levelling the skin surface by smoothing wrinkles or the atrophic scars as well as to model face contours. These liquid substances are administered sub‐ or intra‐dermally and they can temporarily or permanently, fill soft tissue defects. This way, furrows, wrinkles between the eyebrows (glabella), nasolabial folds or mouth corners may be corrected. Collagen is one of the oldest absorbable filler since it is easy to produce, relatively cheap, non‐toxic, non‐carcinogenic, non‐teratogenic, and its usage, ensures reproducible results.^[^ ^328^ ^]^ The second‐generation fillers are mainly, based on HA, which is naturally present in the human body.^[^ ^329^ ^]^ In addition to fillers, other medical devices that are used mainly to improve the cheeks’ appearance and restore the skin volume in the zygomatic area, are the unconventional absorbable threads, made of PLA and glycolic acid or *ε*‐caprolactone. These threads possess microscopic hooks around the circumference, to which they anchor deep into the tissue. As a result, the skin firming effect is visible immediately, after the procedure and the thread remains as a scaffold for flabby skin for many months.^[^ ^330^, ^331^, ^332^ ^]^

## 4 Medical imaging techniques

In medicine, following the *primun non nocere* axiom, non‐invasive medical imaging has always been preferred over invasive imaging. However, whenever a metallic prosthesis is in place (e.g., metallic stents), X‐ray based imaging, namely computed tomography (CT), or magnetic resonance imaging (MRI) is severely limited due to artifacts derived from the metallic structure. For this reason, in the vascular field vessel lumen evaluation through invasive angiography, is still the golden standard for imaging. Beyond angiography, new techniques with high resolution such as intravascular ultrasound or optical coherence tomography, have attained resolutions up to a hundred microns. However, no matter how much these techniques are refined, the instrumentation of the vessel is still required. On the other hand, the use of bioresorbable scaffolds (beyond its unclear clinical benefit over metallic stents at this moment) will clear‐up the path for non‐invasive imaging, since the polymers used will produce none or minimal artifacts to X‐ray or MRI. Therefore, the use of non‐metallic materials conveys this ancillary advantage over metallic materials.

### 4.1 Medical examination of bioresorbable devices

**Figure 9:**
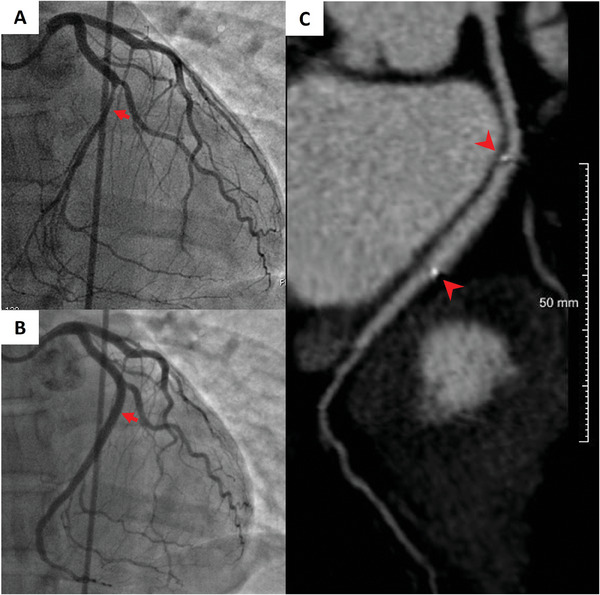
Follow‐up evaluation with CTA of Absorb BVS treatment of a spontaneous coronary dissection. Intracoronary hematoma was observed in the left circumflex coronary artery (Panel A, arrow) and treated with a BRS thereby, achieving a complete recovery of the coronary lumen (Panel B, arrow). CTA could correctly locate the previously implanted stents through the marker identification (Panel C, arrowheads) and confirm good result persistence.

Some studies have explored the ability of computed tomography angiography (CTA) to evaluate bioresorbable scaffolds. Since it was the first platform used in clinical practice, most of the evidence comes from polymeric everolimus‐eluting bioresorbable vascular scaffolds (Absorb BVS, Abbott Vascular). This technology was extensively evaluated with multi‐modality imaging, including for the first time, the CTA as a primary imaging tool for stents. Firstly, a case series,^[^ ^333^ ^]^ reported that all the stented segments were evaluated, and restenosis could be ruled out with a 320‐row CT. Thereafter, the first clinical trial (Absorb A),^[^ ^334^ ^]^ showed that a wide variety of CT scanners could confirm the patency of stents as well as assess the healing process of the vessel wall at 18 months and 5 years follow‐ups. The Absorb II trial^[^ ^335^ ^]^ confirmed the high diagnostic performance of 64‐slice CT scanners and beyond in order to rule out any significant (>50%) in‐stent restenosis and to assess the luminal dimensions when compared to the invasive coronary angiography (ICA) and the intravascular ultrasound at a 3‐year follow‐up in a larger population. Our group has also obtained the same results in an unpublished series of spontaneous coronary dissections that were treated with Absorb‐BVS (**Figure** **9**).

**Figure 10:**
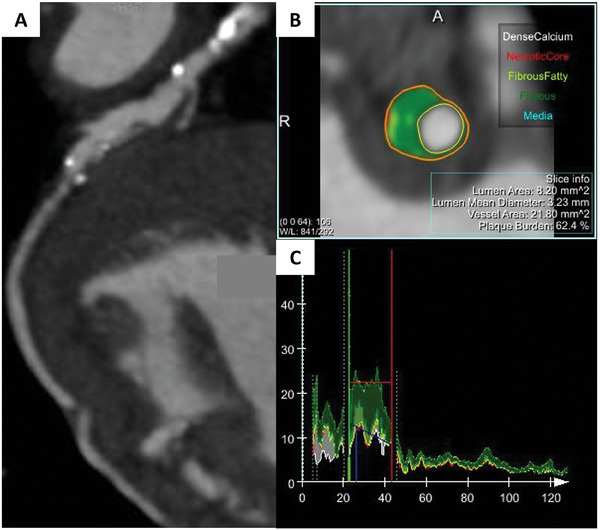
Coronary wall evaluation of a Magmaris bioresorbable scaffold that was implanted in a left circumflex with mixed plaque. Curved multiplanar reconstruction (Panel A) allows stent identification through radiopaque markers and plaque visualization, Plaque analysis tools identify the vessel wall composition at cross‐section (Panel B) and along the vessel (Panel C).

CTA evaluation of the bioresorbable scaffold (**Figure** **10**) permits the identification of luminal contours, hence, providing a valid angiographic assessment when compared to metallic stents.^[^ ^336^ ^]^ Additionally, patients are more receptive to this evaluation when compared to repeated coronary angiography because it is non‐invasive. Moreover, the functional side of coronary artery disease has regained relevance in clinical decision‐making, by frequently using the fractional flow reserve (FFR) as a gatekeeper for revascularization in stable patients. CTA also developed the technology to derive FFR from non‐invasive imaging,^[^ ^337^, ^338^ ^]^ with bioresorbable stents allowing for a full anatomical and functional reconstruction of the vessel. In summary, CTA assessment conveys high diagnostic accuracy, the added value of functional evaluation, patient convenience, and safety, which are factors that should be considered when deciding on the preferred imaging technique for research or clinical purposes.

### 4.2 Requirements and acquisition parameters

Although there are no specific recommendations for bioresorbable stent evaluation with computed tomography angiography (CTA), some lessons learned from conventional scaffold imaging could be extrapolated.^[^ ^339^ ^]^ The image quality derived from the cardiac computed tomography angiography (CCTA) relies on three cornerstones, *viz*: data acquisition, reconstruction, and image display. It is crucial to reduce the motion artifacts that could aggravate blooming artifacts. The patient should be properly and adequately prepared to get a low heart rate (ideally <65 beats per minute) by using the beta blocker drugs, if necessary. Thereafter, the most adequate mode of acquisition is chosen, based on the heart rhythm. The field of view should be adjusted to maximize the number of pixels that are devoted to the depiction of the heart. The slice width should be reduced to the minimum to obtain a greater spatial resolution. A sharp reconstruction kernel is desirable to reduce blooming and increase the edge definition at the cost of higher image noise. Dual‐source CTs are also advisable because they improve the temporal resolution and reduce beam‐hardening artifacts. With regards to the image display, several commercially available software for post‐processing can analyze and quantify the coronary plaque composition.

As per the other imaging modalities, no specific adjustments are needed for bioresorbable scaffold imaging. Angiography will produce a luminogram irrespective of the stent implanted which remains within the vessel wall limits. The metallic stents will be easily seen whereas bioresorbable scaffolds are invisible to the standard angiography and therefore are usually enhanced with metallic markers at the edges or iodine compounds to facilitate visualization. Stent or scaffold enhancing software such as Stent Boost (© Phillips), or ClearStent (©Siemens), may facilitate the visualization of metallic structures or scaffold markers. In intravascular ultrasound metal usually leaves an acoustic shadow behind which is not seen with polymeric scaffold struts, thus being easily differentiated with standard imaging settings. Very similarly, light‐based optical coherence tomography will have a reflection artifact with a shadow behind the metallic strut, whereas light can go across the polymeric structure, hence allowing for in vivo measurement of the strut thickness, potential separation from the vessel wall, and even behind‐the strut analysis of the vessel wall, again with standard settings of the imaging system.

### 4.3 Bench‐testing and non‐clinical use of medical imaging

Different from the human use, micro‐computed tomography (micro‐CT) has great resolution, thanks to the unlimited threshold for radiation and the lack of movement of the sample, which leaves behind, many acquisition problems and artefacts, derived from cardiac motion. Micro‐CT is capable of very high resolution, up to ≈1 µm per voxel and it allows for the quantitative and qualitative analyses and three‐dimensional reconstructions.

Bifurcation models are commonly used to test different PCI techniques that usually require extensive manipulation of the stent platform. BRS are, however, more fragile than metallic stents, and the BRS integrity is paramount due to clinical findings of BRS collapse, in cases of strut fracture during implantation. Although it might be detected by intracoronary imaging assessment (OCT), only with the micro‐CT a comprehensive assessment can be made, thanks to the volumetric three‐dimensional acquisition. Bifurcation treatment with a single stent or scaffold requires the device to fit a distal diameter of ≈0.678 of proximal diameter (Finet's simplification of Murray's fractal law^[^ ^340^ ^]^). This step‐up in diameter must be adequately reproducible by the device without malposition (separation from vessel wall) or recoil (elastic resistance to vessel dilatation).

The absorb BRS platform was tested for integrity after opening a lateral cell to improve the flow to a simulated side branch and re‐expanding of the proximal part of the BRS, showing similar results as the standard DES when compared with the micro‐CT. With a threshold of 1 mm step‐up in diameter, the BRS showed a good adaptation to the fractal geometry without a strut fracture.^[^ ^341^ ^]^

The Fantom Encore scaffold (REVA Medical, San Diego, California, US), a new generation of the BRS, made of iodinated polycarbonate copolymer of tyrosine analogue and biocompatible hydroxyesters, was tested in a coronary bifurcation bench model to observe the expansion capacity. Fourteen scaffolds were tested in two different bench models of an elastomeric material,^[^ ^342^ ^]^ with the micro‐CT quantitative analysis. The study found 1 (0.7%) broken ring in the small vessel model and 14 (10%) broken rings for the large platforms.

### 4.4 Future directions of medical imaging

The ongoing medical imaging development relies on three cornerstones, *viz*: image quality improvement, reduction in data acquisition time and automation of image interpretation. The first two aspects are closely related to technical advances. Materials technology has allowed the miniaturization of imaging devices and components that have been reflected in gains in terms of spatial resolution, while the revolutions in computer processing, are the basis of the acceleration in image acquisition. Consequently, temporal resolution has been improved and more patients may benefit from their access to non‐invasive imaging tests. The last condition has been boosted by the growth of artificial intelligence (AI), which has shortened the study of reading through anatomical structure identification and automatic measurements.

In particular, the following steps in CCTA evaluation of bioresorbable scaffolds will rely on different fronts, i.e., the technological development of image acquisition, new tools of functional evaluation and support of AI for image postprocessing and analysis. Some of the advances in technology have been mentioned previously. Probably the most relevant issue in stent evaluation is to maximize the spatial resolution. The ongoing movement in this direction is the appearance of photon‐counting scanners. However, they have some relevant drawbacks that need to be resolved. The main amelioration is to have a widely available advanced hardware to manage the large number of images that are generated with this technique. The other key progress is the expansion of the tools for functional assessment of the coronary stenosis, with the use of CCTA. There are two ongoing approaches: FFR and myocardial perfusion imaging (MPI). The FFR‐CT has already been proven in native coronary atherosclerosis with a diagnostic performance that is close to the invasive FFR. However, the suboptimal visualization of the coronary lumen in the stented segments has precluded its use in patients with previous percutaneous coronary intervention (PCI). In contrast, bioresorbable stents do not have this limitation and, therefore, could potentially be evaluated with FFR‐CT. Nevertheless, the accuracy of this technique needs to be proven. MPI‐CT does have data regarding the conventional stent evaluation, showing similar diagnostic performance to an invasive FFR, but the bioresorbable scaffolds are yet to be evaluated with this technique. Moreover, although associated radiation has already been reduced, acquisition protocols should be further simplified to spread its clinical use.

Finally, the AI blooming has raised an interest in the field of medical imaging. Some initial advances are already available for simple tests, e.g., the X‐rays diffraction technique. However, there is still some controversy regarding the clinical applicability of this “computing revolution”. AI is making ongoing changes, viz handling a vast amount of data storage scenario, de‐noising imaging, iterative coronary motion compensation, automated coronary extraction and segmentation, accurate stenoses evaluation, and the quantification and characterization of atherosclerotic plaque, with CTA acquisition and interpretation.^[^ ^343^ ^]^ On the other hand, today's AI is probably, not as revolutionary as claimed, and it raises some concerns about its: reproducibility, relevance in real‐world clinical practice, data ownership, and ethical‐legal considerations.^[^ ^344^ ^]^

## 5 Regulatory Challenges in Translating Research into Clinics

At a certain stage in the development of each medical device, it will have to be introduced into the market. World institutions and organizations create regulations to establish high quality and safety standards and harmonize the rules that allows medical devices in the market. In May 2021, the European Union implemented the Medical Device Regulation 2017/745 (MDR) establishing its definition, thus: “*as any instrument, apparatus, appliance, software, implant, reagent, material or other article intended by the manufacturer to be used, alone or in combination, for human beings for one or more of the following specific medical purposes*”.^[^ ^345^ ^]^ Resorbable polymer‐based biomaterials fall into this definition, therefore if they are to be introduced into the market, they must meet the legal requirements to ensure that they are safe and perform as intended. The first step is to categorize the medical device into one of three classes, according to the level of harm/discomfort that it may pose to users or patients, from the least invasive class I, including sterile and measuring devices, through class II bearing medium (class IIa) or medium‐high risk (class IIb) to the high‐risk devices classified as III. The classification of a medical device determines the type of conformity assessment procedure to be performed by the manufacturer to finally place a CE mark (*Conformité Européenne*) on a device. The higher the product class, the more restrictive the procedure. The conformity assessment usually involves an audit of the manufacturer's quality system and, depending on the type of device, a review of the technical documentation from the manufacturer on the safety and performance of the device must be carried out. For the non‐sterile and non‐measuring medical devices of the first class (class I), the manufacturer carries out the conformity assessment, issues a declaration of conformity and marks their device with the CE mark. For products of the IIa, IIb, and III classes, a notified institution must participate in the procedure. The last step is to register the product with the Competent Authority.

**Figure 11:**
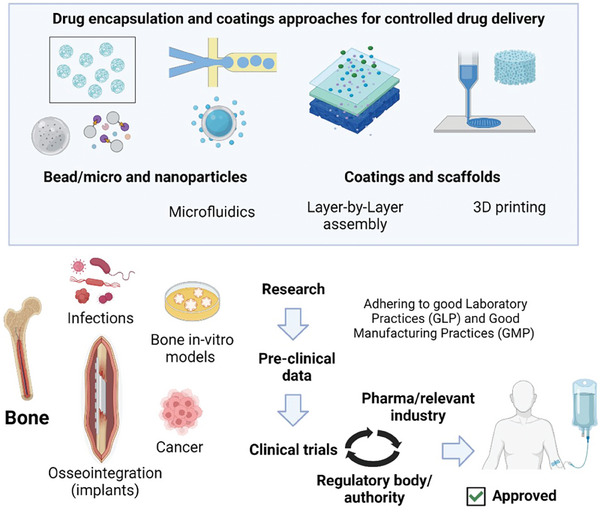
Schematic representation of the different approaches that are being exploited for controlled drug delivery in different bone applications and a brief regulatory pathway for approval toward clinics.

The assignment of a product into a specific class is not straightforward. Resorbable polymeric‐based products, such as absorbable sutures, hydrogel wound dressing, or standardized and customized implants, fall within the majority of the classes depending on the degree of their invasiveness, intended duration of use, and the extent of their contact with the human body.^[^ ^346^ ^]^ In some cases, when a medicinal product is added to the bioresorbable formulation, like in the case of drug delivery systems, the medical device is then excluded from the Directives by their scope. Therefore, it is classified as a borderline product and falls within rigorous regulations of medicinal products, especially in terms of its complying with multistage clinical trials.^[^ ^347^ ^]^ Addressing these challenges, requires a close collaboration between the researchers, clinicians, and the regulatory agencies to ensure that the resorbable materials meet the necessary standards for safety and efficacy before allowing their entering into the market. The development of advanced drug delivery platforms and precision medicines for regulatory bodies, e.g., the European Medicines Agency (EMA), Medicines, and Healthcare Products Regulatory Authority (MHRA), FDA, etc., the approval and clinical usage protocols, present formidable regulatory challenges, particularly when it comes to drug delivery strategies for treating various bone‐related conditions (**Figure** **11**). Indeed, the regulation of nanomedicines‐based products and drug delivery systems, differ between countries and regions, and therefore, researchers are encouraged throughout the development process, to communicate with the relevant regulatory bodies for the targeted market.^[^ ^348^ ^]^ Some of the general challenges that relate to the translation of drug delivery, encompasses their properties (physical, chemical, biological, e.g., pharmacodynamic and pharmacokinetic profiles, which may differ from their constituent materials and payloads), activity batch consistency, and reproducibility of the manufacturing, for scale‐up purposes. The following are some of the specific challenges that are associated with bone infections, bone cancer, promoting osseointegration, and validating models.

The regulatory hurdles arise in the demonstration of the safety and efficacy of the drug delivery systems for the treating of bone infections. Complex interactions between the drug, biomaterial carrier, and the host immune response^[^ ^349^ ^]^ necessitate comprehensive pre‐clinical studies. The establishment of an appropriate animal model that mirrors human infection scenarios, while adhering to the regulatory standards, can be challenging. Additionally, ensuring a controlled and sustained drug release to combat infection, while minimizing systemic effects, poses a delicate balance. The development of drug delivery systems for bone cancer requires extensive pre‐clinical evaluation that encompasses safety, bio‐distribution, and therapeutic efficacy. Clinical translation demands comprehensive understanding of the systemic drug exposure, potential off‐target effects, and long‐term outcomes. Rigorous toxicology studies are essential to assess any adverse effects on the bone and other tissues. Moreover, the demonstration of the potential for controlled and targeted drug release within the tumor micro‐environment, poses intricate challenges. Regulatory approval for drug delivery systems that promote osseointegration, necessitates rigorous demonstration of implant safety, efficacy, and long‐term biocompatibility. Long‐term biocompatibility assessments in relevant animal models are crucial, since the integration process unfolds over extended periods. Ensuring that the drug‐loaded implant does not hinder or impede the natural bone healing process is a critical aspect of the regulatory evaluation. The translation of a high‐throughput drug screening from bone models to clinical applications, requires robust validations. The establishment of the relevance and predictive values of the in vitro and in vivo models for the real‐world outcomes, is a challenging task. Striking a balance between the model complexity and the throughput efficiency, while demonstrating good reproducibility and clinical correlation, demands meticulous experimental design and rigorous validation studies.^[^ ^350^ ^]^

In all these scenarios, the regulatory agencies therefore demand comprehensive evidence of safety, efficacy, and clinical relevance. Adherence to Good Laboratory Practices (GLP) and Good Manufacturing Practices (GMP) is vital, thereby ensuring that research processes and manufacturing standards adhere to the regulatory guidelines. Addressing the specific requirements for each application, such as localized drug release, biocompatibility, and long‐term effects, is a complex undertaking. A successful translation hinges on the interdisciplinary collaboration between researchers, clinicians, regulatory experts, and the industry partners. A close alignment with the regulatory agencies throughout the research process aids in anticipating and addressing challenges discussed earlier. While navigating these regulatory challenges is demanding, the potential to positively impact patient lives, through innovative drug delivery strategies in bone‐related conditions, remains an inspiring pursuit.

## 6 Market Potential

The market for resorbable materials used in dental and bone clinical applications is expected to grow significantly. For instance, the resorbable implants market is estimated to reach $11.5 billion by 2027 and it is poised to grow at a CAGR of 7.6% between 2022 and 2027. Based on the material type, the resorbable implants market can be further segmented, into PGA, PLA, PCL, PLGA, polydioxanone, PLLA, and poly‐beta‐hydroxybutyrate. PLLA held the dominant market share in the year 2021, since it is a biopolymer that is easy to produce and is biodegradable, and it is made from abundant renewable resources. Based on the current market trends, the senior population is expected to grow, and researchers predict that this population, particularly those over 65 years, will undergo surgeries in areas such as hip replacement, knee replacement, shoulder replacement, and spinal surgery. Resorbable implants are commonly used in orthopedic treatments, therefore, this trend is expected to benefit the market.^[^ ^351^ ^]^

**Figure 12:**
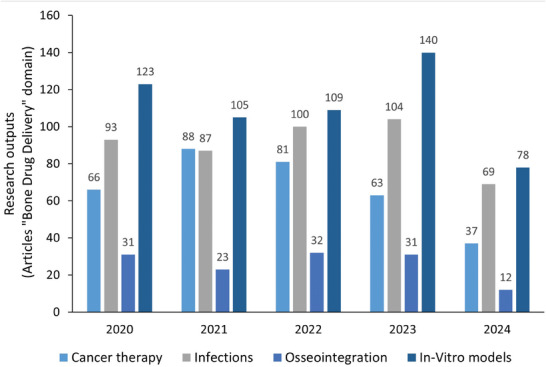
Scholarly research outputs in the last 5 years, in “Bone Drug Delivery” domain in the fields of cancer therapy, infection management, osseointegration and bone in vitro *models*. Data extracted from Web of Science (accessed August 2^nd^, 2024, research articles excluding review articles and book chapters).

According to recent reports, the market for the drug delivery systems had a value of USD 39.55 billion in the year 2022, with a projected increase to USD 42.71 billion in 2023. Furthermore, it is expected to grow even more, and it is estimated to reach a value of USD 78.76 billion by the year 2030, with a compound annual growth rate of ≈9.1% during the forecast period.^[^ ^352^ ^]^ Consequently, there has been a significant growth in the market and in research of bone cancer therapy and infection management (**Figure** **12**). Targeted drug delivery systems have become popular in bone cancer therapy with a focus on improving the patient outcomes and minimizing the associated side effects, particularly the use of nanoparticles.^[^ ^353^ ^]^ Personalized medicine has also contributed to the demand for customized drug delivery strategies. Research in antibiotic‐loaded biomaterials and smart delivery systems has increased due to the urgent need for effective infection management. This segment highlights the potential for growth, especially with the rise of implant‐associated infections. The market for osseointegration has expanded with advancements in orthopedic and dental implant technologies.^[^ ^354^ ^]^ Therefore, researchers are exploring new drug delivery methods^[^ ^281^ ^]^ in order to enhance implant success rates and patient satisfaction in the long term. Advanced bone models for drug testing are important for efficient pre‐clinical evaluations, and the market for such models is expected to grow, since there is a demand for the testing platforms that are predictive, efficient, and reduce animal usage, while accelerating drug development. The dynamic growth of drug delivery markets in bone reflects the increasing emphasis on patient‐centered solutions, spurring innovative research and fostering collaborations between clinicians, researchers, and the pharmaceutical industry.

## 7 Conclusions and Future Trends

This article has provided a vivid overview of recent advances in resorbable biomaterials and composites, focusing on their geometric forms and their applications in tissue engineering, drug delivery, dental, and cardiovascular fields. The engineering approaches for the fabrication of resorbable composite scaffolds and stents, vascular grafts, as well as cardiac patches, offer clinical insights, needed for the improvement of the material characteristics, are also provided. Additionally, the studies that entail the use of cardiac computer tomography to evaluate the internal structures of bioresorbable scaffolds and stents, which emphasize the need for strategies required for the design of multifunctional resorbable implants for specific biomedical needs are summarized.

The future trends that are expected in tissue engineering, particularly when it involves bioresorbable polymers, are poised to experience desirable and significant advancements that will be driven by innovations in material science and biotechnology. Advanced bioresorbable polymers are expected to exhibit enhanced mechanical properties, biocompatibility, and should be tailored for appropriate degradation profiles, that can mimic closely, the natural ECM to facilitate efficient tissue regeneration. Hybrid scaffolds that integrate bioresorbable polymers with various fillers are expected to offer adequate mechanical support and at the same time, deliver therapeutic agents directly to the site of tissue repair, thereby enhancing the healing process. The emerging fabrication technologies, such as 3D printing and electrospinning, are anticipated to enable the creation of complex, patient‐specific scaffolds with precise pore architecture that can improve tissue integration and vascularization. Personalized medicine is expected to benefit from the patient‐specific scaffolds based on the individual anatomical data, hence, leading to customized treatments with improved precision and effectiveness. Regulatory and clinical advancements will be crucial for the adoption of these envisaged innovations. These should be accompanied with standardized testing protocols and regulatory approvals by dedicated agencies that will facilitate their translation into clinical practice. Comprehensive clinical trials and long‐term studies are necessary to validate the safety and efficacy of these materials.

In summary, the future of tissue engineering with bioresorbable polymers is highly promising, with dedicated advancements in material design, fabrication techniques, and personalized medicine that will lead to more effective and tailored treatments, with anticipated improved patient outcomes, and broad applications in regenerative medicine.

## Conflict of Interest

The authors declare no conflict of interest.

## Data Availability

The data described in the article are available at https://zenodo.org/records/13318550. We would appreciate if other researchers could benefit from our literature and results. This will foster discussions and collaboration among scientists worldwide.
